# Proteomics Identifies Circulating TIMP-1 as a Prognostic Biomarker for Diffuse Large B-Cell Lymphoma

**DOI:** 10.1016/j.mcpro.2023.100625

**Published:** 2023-07-26

**Authors:** Ning Lou, Guibin Wang, Yanrong Wang, Meng Xu, Yu Zhou, Qiaoyun Tan, Qiaofeng Zhong, Lei Zhang, Xiaomei Zhang, Shuxia Liu, Rongrong Luo, Shasha Wang, Le Tang, Jiarui Yao, Zhishang Zhang, Yuankai Shi, Xiaobo Yu, Xiaohong Han

**Affiliations:** 1 Department of Clinical Laboratory, National Cancer Center/National Clinical Research Center for Cancer/Cancer Hospital, Chinese Academy of Medical Sciences & Peking Union Medical College, Beijing Key Laboratory of Clinical Study on Anticancer Molecular Targeted Drugs, Beijing, China; 2 State Key Laboratory of Proteomics, Beijing Proteome Research Center, National Center for Protein Sciences-Beijing (PHOENIX Center), Beijing Institute of Lifeomics, Beijing, China; 3 Department of Medical Oncology, National Cancer Center/National Clinical Research Center for Cancer/Cancer Hospital, Chinese Academy of Medical Sciences & Peking Union Medical College, Beijing Key Laboratory of Clinical Study on Anticancer Molecular Targeted Drugs, Beijing, China; 4 Clinical Pharmacology Research Center, Peking Union Medical College Hospital, State Key Laboratory of Complex Severe and Rare Diseases, NMPA Key Laboratory for Clinical Research and Evaluation of Drug, Beijing Key Laboratory of Clinical PK & PD Investigation for Innovative Drugs, Chinese Academy of Medical Sciences & Peking Union Medical College, Beijing, China

**Keywords:** antibody microarray, diffuse large B-cell lymphoma, mass spectrometry, proteomics, prognostic biomarker

## Abstract

Diffuse large B-cell lymphoma (DLBCL) is a heterogeneous disease, although disease stratification using in-depth plasma proteomics has not been performed to date. By measuring more than 1000 proteins in the plasma of 147 DLBCL patients using data-independent acquisition mass spectrometry and antibody array, DLBCL patients were classified into four proteomic subtypes (PS-I-IV). Patients with the PS-IV subtype and worst prognosis had increased levels of proteins involved in inflammation, including a high expression of metalloproteinase inhibitor-1 (TIMP-1) that was associated with poor survival across two validation cohorts (n = 180). Notably, the combination of TIMP-1 with the international prognostic index (IPI) identified 64.00% to 88.24% of relapsed and 65.00% to 80.49% of deceased patients in the discovery and two validation cohorts, which represents a 24.00% to 41.67% and 20.00% to 31.70% improvement compared to the IPI score alone, respectively. Taken together, we demonstrate that DLBCL heterogeneity is reflected in the plasma proteome and that TIMP-1, together with the IPI, could improve the prognostic stratification of patients.

## Highlights

• Proteomic platform combining DIA-MS and antibody array quantifies 1000+ plasma proteins.
• Plasma proteome classifies DLBCL into four subtypes with different prognosis.
• High TIMP-1 level shint poor prognosis, could complement IPI score for risk stratification.

## Graphical Abstract

**Figure:**
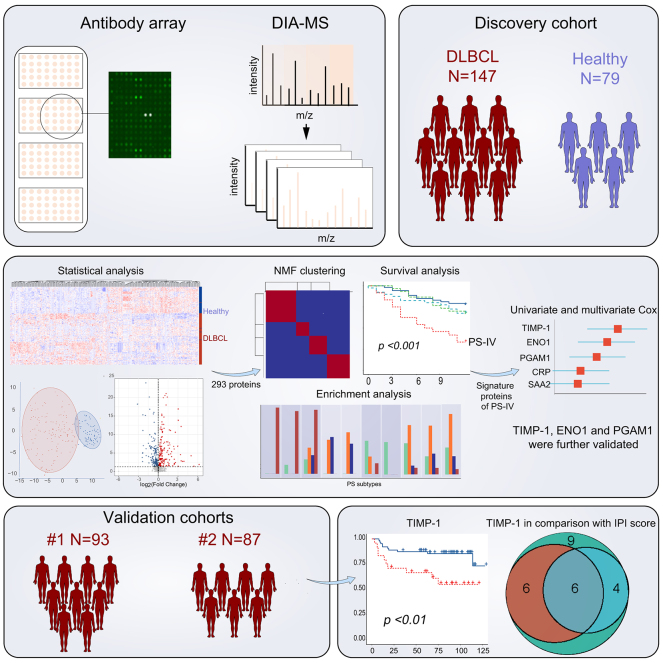

## In Brief

The in-depth proteomic platform quantifies more than 1000 proteins and classifies the DLBCL patients into four subtypes with distinct prognosis. Among signature proteins of PS-IV subtype with the worst prognosis, TIMP-1 was identified to be associated with poor prognosis and validated in two independent cohorts, which may help improve the prognostic ability of the IPI score for R-CHOP treatment.

Diffuse large B-cell lymphoma (DLBCL) is the most common type of non-Hodgkin lymphoma, which is highly heterogeneous and accounts for the majority of newly diagnosed non-Hodgkin lymphoma cases in the United States and China (1, 2). Currently, the international prognostic index (IPI) is implemented widely in the clinic to predict 5-year overall survival (OS). This scoring system based on age, Ann Arbor stage, serum lactate dehydrogenase concentration, performance status, and the number of extranodal disease sites is separated into four risk categories: low (IPI = 0–1; 5-year OS = 73%), low intermediate (IPI = 2; 5-year OS = 51%), high intermediate (IPI = 3; 5-year OS = 43%), and high (IPI = 4–5; 5-year OS = 26%). In the last 20 years, significant efforts have been made to decode the molecular heterogeneity of DLBCL using genomics and transcriptomics and to develop new methods to predict clinical outcomes (3, 4, 5, 6, 7, 8).

In 2000, Alizadeh *et al.* analyzed the gene expression of lymphocyte subpopulations in 96 patients with DLBCL using DNA microarrays. Two DLBCL molecular subtypes were identified, which included a germinal-center B-cell-like (GCB) subtype that resulted in a better prognosis than the activated B-cell-like subtype (3). Using the same technology to analyze 240 DLBCL patients, Rosenwald *et al.* (6) identified three disease subtypes, including GCB, activated B-cell-like, and type 3 diffuse large B-cell lymphoma (type 3). The GCB subtype, which contains oncogenic BCL-2 translocation and c-REL amplification, was associated with the highest 5-year survival rate. Finally, a 17-gene signature independent of the IPI was developed to predict the prognosis of DLBCL patients after chemotherapy.

In 2020, Wright *et al.* developed a LymphGen algorithm that classified DLBCL patients into seven genetic subtypes using genomic and transcriptomic data from three sample cohorts (National Cancer Institute; Harvard Medical School; BC Cancer Agency) (4, 7, 9). Interestingly, the EZB subtype that had highly expressed genes related to B-cell differentiation was associated with a good prognosis. In contrast, the N1 subtype with highly expressed genes involved in oncogenic signaling pathways and tumor microenvironment was associated with a worse prognosis (8). These results highlight the value of genomics and transcriptomics in identifying patients who would benefit the most from the R-CHOP (rituximab plus cyclophosphamide, doxorubicin, vincristine, and prednisone) treatment regimen. However, previous studies of molecular subtypes using complementary DNA microarrays and whole exome sequencing are time-consuming (∼11–16 weeks) (10), and the availability of sufficient fresh tissue for RNA extraction was difficult.

To address this issue, Hans *et al.* (5) successfully reproduced the classification of GCB and non-GCB subtypes by immunostaining five proteins (CD10, BCL-6, MUM1, cyclin D2, BCL-2) in 152 DLBCL patients. Their work demonstrates the possibility of using immunostaining to classify DLBCL and predict patient outcomes. The above classification methods are summarized in supplemental Table S1. While Hans classification system using immunostaining is easier (5), it is expensive, not reproducible across laboratories, and not commercially available (11). Thus, a new classification system that is accurate and easy to use is needed.

Plasma (or serum) contains thousands of circulating proteins that are produced by different organs and play important roles in modulating humoral immunity, inflammation, coagulation, and metabolism. Moreover, the expression changes of plasma proteins reflect the dynamism of human physiology and pathology. As such, the comprehensive profiling of plasma proteome is valuable in elucidating disease mechanisms, identifying potential biomarkers for diagnosing disease, and monitoring the response to treatment (12, 13, 14, 15).

In this work, we systematically characterized DLBCL molecular heterogeneity of 147 DLBCL patients and 79 healthy controls (HCs) by performing in-depth analyses of the plasma proteome using data-independent acquisition mass spectrometry (DIA-MS) and customizable antibody microarrays (16, 17). Using non-negative matrix factorization (NMF) analysis, the DLBCL patients could be classified into different proteomics subtypes with distinct biological processes. Moreover, the clinical utility of proteomics subtypes in predicting the prognosis of DLBCL patients treated with R-CHOP or R-CHOP–like regimens was investigated. Predictive biomarkers of a poor prognosis such as metalloproteinase inhibitor-1 (TIMP-1) in a particular DLBCL subtype were validated in two independent cohorts (n = 180) and compared with the current IPI score classification system.

## Experimental Procedures

Reagents and Tools table

**Table:** The reagents and tools used in this study

Reagent/resource	Reference or source	Identifier or catalog number
Experimental models		
Blood plasma samples (*Homo sapiens*)	This study	N/A
Chemicals, enzymes, and other reagents		
NHS-PEG4-Biotin	Thermo Fisher Scientific	Cat #21330
Urea	Sigma-Aldrich	U5378-500G
DTT	Sigma-Aldrich	A620058-0025
Iodoacetamide (IAA)	Sigma-Aldrich	A600539-0005
Formic acid	Sigma-Aldrich	Cat # 64-18-6
Trypsin	Promega	20210810C
Acetonitrile	Thermo Fisher Scientific	185439
Water, LC/MS Grade	Thermo Fisher Scientific	0000250623
Software		
GenePixPro7	Molecular Devices	N/A
Spectronaut pulsar × 12.0	Biognosys	N/A
Human FASTA database	https://www.uniprot.org/	N/A
R(4.0.2)	https://www.R-project.org/.	N/A
PANTHER database	http://pantherdb.org/	N/A
Omicsbean	http://www.omicsbean.cn/	N/A
Cytoscape (3.7.2)	http://www.cytoscape.org	N/A
ClueGO	https://apps.cytoscape.org/apps/cluego	N/A
Cytohubba	https://apps.cytoscape.org/apps/cytoHubba	N/A
STRING	https://cn.string-db.org/	N/A
Hiplot	https://hiplot.com.cn/	N/A
Real Statistics Using Excel	https://www.real-statistics.com/non-parametric-tests/mann-whitney-test/mann-whitney-power/	N/A
Other		
Genepix 4300A microarray scanner	Molecular Devices	141095
Thermo Q Exactive HF mass spectrometer	Thermo Fisher Scientific	Cat # IQLAAEGAAPFALGMBFZ
Eppendorf refrigerated centrifuge	Eppendorf	5810R
PGAM1 ELISA Kit	mlbio	ml901164
ENO1 ELISA Kit	mlbio	ml038486
TIMP-1 ELISA Kit	Sino Biological	KIT10934

### Methods and Protocols

#### Clinical Samples

The clinical samples were collected between 2010 and 2019 (discovery cohort: 2010–2016, validation cohorts: 2010–2019, HCs: 2015–2018). All plasma samples collected in this study underwent the same procedure according to the Clinical Diagnosis and Management by Laboratory Methods [John Bernard Henry, SAUNDERS Company (20th Edition), 2001]. Blood was drawn into a vacutainer tube with a lavender cap that contained the anticoagulant EDTA. Preprocessing holding time was within 12 h, and the temperature was maintained at 4 °C to ensure minimal effects on the plasma proteome when compared to samples that are processed immediately after collection (18). Tubes were centrifuged for 10 min at 2000*g*, 20 °C, and then transferred to a sterile 2 ml conical tube. The samples were stored at −80 °C prior to proteomics measurement without repeated freeze-thaw cycles. Totally, 200 μl plasma was used for DIA-MS and antibody array experiments. All samples from DLBCL patients were collected prior to treatment.

There were three patient cohorts in this study. The discovery cohort was comprised of 147 DLBCL patients and 79 HCs (supplemental Table S2). The validation cohort #1 consisted of 93 DLBCL patients and the validation cohort #2 contained 87 DLBCL patients (supplemental Table S2). All patient samples were collected before CHOP, CHOP-like, R-CHOP, or R-CHOP–like chemotherapy treatment where “R” represents the monoclonal antibody rituximab. Immunohistochemistry results for CD10, bcl-6, and MUM1 were used to determine GCB and non-GCB groups. If CD10 alone was positive, cases were assigned to the GCB group or if both bcl-6 and CD10 were positive. If both bcl-6 and CD10 were negative, the case was assigned to the non-GCB subgroup. If bcl-6 was positive and CD10 was negative, the expression of MUM1 determined the group; if MUM1 was negative, the case was assigned to the GCB group; if MUM1 was positive, the case was assigned to the non-GCB group (5). Patients who met any of the following criteria were excluded from the study: primary central nervous system lymphoma; patients who received less than three chemotherapy cycles of standard CHOP, CHOP-like, R-CHOP, or R-CHOP-like regimen; or patients with a history of another primary malignancy within 5 years of DLBCL diagnosis. Participants in the HC group did not have any malignant tumors or history of malignant tumors. Any participants classified as an HC were excluded from this study if their tumor markers, liver function, or routine blood tests (*e.g.*, C-reactive protein, CRP) were flagged as outside the normal "healthy" range. The assembled HC cohort included 79 HCs who matched in age and gender with 147 DLBCL patients. In addition, the liver and kidney functions of DLBCL patients and HCs did not differ (supplemental Fig. S1).

The baseline characteristics of the three patient cohorts are shown in Table 1. The treatment efficacy was defined as complete response (CR), partial response, stable disease, or progressive disease according to the Response Evaluation Criteria in Solid Tumours version 1.1 for lymphoma. Responder (R) was defined as CR to front-line treatment without disease progression, and non-responder (NR) was defined as disease progression after a CR or not reaching CR to front-line treatment. The follow-up visit was performed annually by a callback to inquire about the progression and survival of DLBCL patients from the time of the first treatment to December 31, 2019. This study was approved by the Ethics Committee of the Cancer Hospital of the Chinese Academy of Medical Sciences (19-019/1804). The study was executed according to the principles of the Declaration of Helsinki, and since the plasma samples used in this study were leftovers of routine clinical tests, so waivers of informed consent were requested.

**Table 1:** Clinical characteristics of patients who provided samples for this study

Clinical characteristics	Discovery cohort		Validation cohort 1	Validation cohort 2
	DLBCL (n = 147)	HCs (n = 79)	DLBCL (n = 93)	DLBCL (n = 87)
Chemotherapy				
R-CHOP/R-CHOP-like treatment	129 (87.8)	/	93 (100)	87 (100)
CHOP/CHOP-like treatment	17 (11.5)	/	0 (0.0)	0 (0.0)
hyper-CVAD	1 (0.7)	/	0 (0.0)	0 (0.0)
Gender				
Female, n (%)	68 (46.3）	36（45.6）	36 (38.7)	43 (49.4)
Male, n (%)	79 (53.7）	43（54.4）	57 (61.3)	44 (50.6)
Age, years				
≤60, n (%)	94 (63.9）	56（70.9）	32 (34.4)	52 (59.8)
>60, n (%)	53 (36.1）	23（29.1）	61 (65.6)	35 (40.2)
Cell of origin (Hans classification)				
GCB, n (%)	36 (24.5）	/	39 (41.9)	30 (34.5)
Non-GCB, n (%)	111 (75.5）	/	44 (47.3)	45 (51.7)
Unknown, n (%)	0 (0.0)	/	10 (10.8)	12 (13.8)
Extranodal involvement				
≤1, n (%)	98 (66.7）	/	57 (61.3)	64 (73.6)
>1, n (%)	41 (27.9）	/	28 (30.1)	23 (26.4)
Unknown, n (%)	8 (5.4）	/	8 (8.6)	0 (0.0)
Ann Arbor stage				
I/II, n (%)	74 (50.3）	/	54 (58.1)	51 (58.6)
III–IV, n (%)	73 (49.7）	/	39 (41.9)	36 (41.4)
IPI score				
0～2, n (%)	101 (68.7）	/	70 (75.3)	66 (75.9)
3～5, n (%)	46 (31.3）	/	19 (20.4)	21 (24.1)
Not reported, n (%)	0 (0.0)	/	4 (4.3)	0 (0.0)

Abbreviation: n, number of patients.

#### Experimental Design and Statistical Rationale

**Fig. 1:**
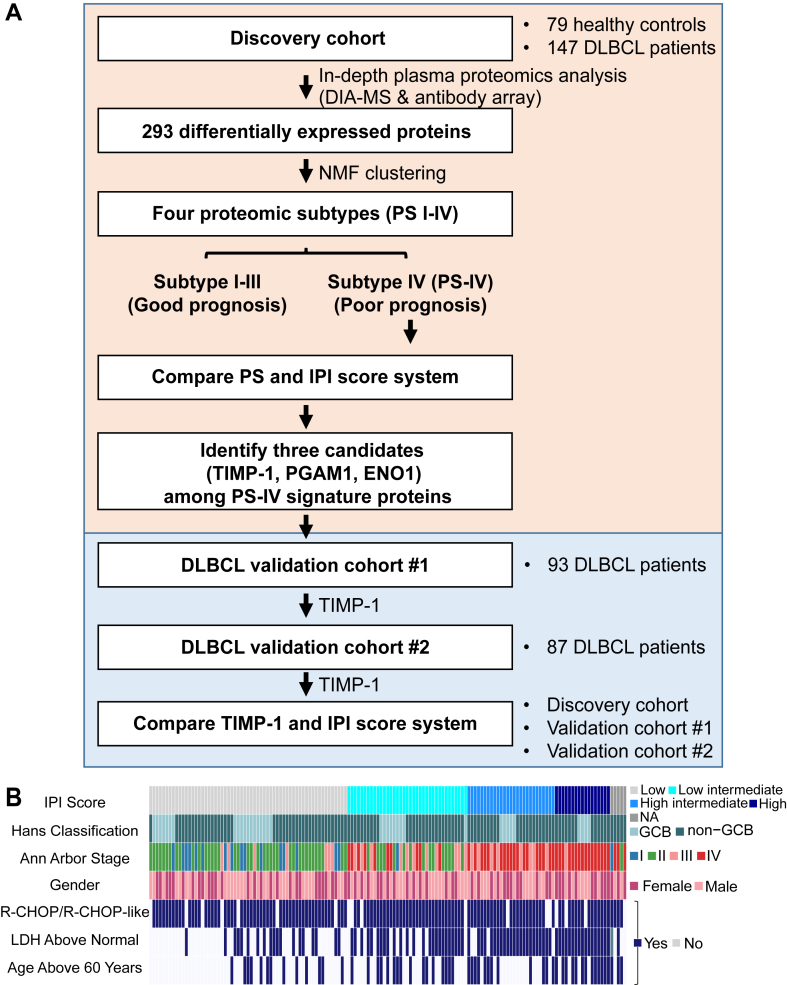
**Plasma protein profiling of DLBCL patients using in-depth proteomics.***A*, schematic overview of the study. *B*, the study cohort included 147 DLBCL patients. Clinical parameters are indicated in the heat map. DLBCL, diffuse large B-cell lymphoma; LDH, lactate dehydrogenase.

The workflow is illustrated in Figure 1. The proteome of the samples was studied by DIA-MS combined with antibody arrays. The reproducibility of DIA-MS and antibody microarray measurements were measured using 20 replicates of HeLa cell lysates and four replicates of quality control plasma samples from HCs, respectively. Pearson's correlation was used to evaluate the repeatability of protein quantification. The analysis of DIA-MS and AA is based on false discovery rate (FDR) value <0.05 as the significant threshold. Protein annotations were analyzed by gene ontology (GO) and Kyoto Encyclopedia of Genes and Genomes (KEGG) databases. NMF was applied to explore the plasma heterogeneity of DLBCL. Metaproteins were identified by NMF algorithm, and prognostic biomarkers were selected from metaproteins by Cox and Kaplan–Meier analysis (*p* ≤ 0.05). The random forest algorithm was performed to identify the proteomics subtype-IV (PS-IV) with the worst prognosis, and decision tree analysis provided a formula that combines TIMP-1 with IPI score.

#### Analysis of DLBCL Plasma Proteome Using Antibody Microarrays

The antibody microarray targeting 551 unique human proteins in duplicate was prepared by ProteomicsEra Medical Co, Ltd as previously described (16, 17, 19). The biotin labeling of plasma proteins was performed as previously described (16, 17). Prior to the assay, the glass-based antibody microarrays were blocked with 500 μl of 5% milk (w/v) in each well for 1 h. After removing the milk, the antibody microarrays were incubated with biotinylated plasma proteins at 4 °C overnight (8 h). After washing the slide three times with PBS containing 5% Tween-20, the array was incubated with 2 μg/ml streptavidin-Cy3 for 1 h at room temperature and then washed three times with PBS containing 5% Tween-20. The fluorescent readout was detected using a GenePix 4300A microarray scanner at a wavelength of 532 nm (Molecular Devices). The signal intensity was extracted with GenePixPro7 software (Molecular Devices) (https://www.moleculardevices.com/products/additional-products/genepix-microarray-systems-scanners). The median pixel intensity of each spot was subtracted by the median pixel intensity of the adjacent background to remove the effect of nonspecific binding or spatial heterogeneity across the array with GenePixPro7 software (Molecular Devices). The average of each protein was then calculated across duplicate spots. If the median pixel intensity of a spot was below the median pixel intensity of the adjacent background, the spot was assigned as the missing value.

#### Analysis of the DLBCL Plasma Proteome Using DIA-MS

##### Sample Preparation

One microliter (1 μl) per sample was mixed with lysis buffer containing 8 M urea; 100 mM Tris–HCl, pH 8.5 (Sigma-Aldrich); and 10 mM DTT, then reduced at 37 °C for 60 min, and alkylated with 50 mM iodoacetamide at room temperature for 30 min in the dark. Protein digestion was performed by the filter-aided sample preparation method with trypsin (Promega) in 50 mM NH_4_HCO_3_ (Sigma-Aldrich) (20). The peptide concentration was detected by a NanoDrop (Thermo Fisher Scientific) at an absorbance of 280 nm.

##### LC MS/MS Analysis

First, a spectral library by data-dependent acquisition (DDA) was constructed for DIA analysis, and mixed peptides from each sample were separated into ten fractions by RIGOL L-3000 HPLC (Puyuan Jingdian Science and Technology Ltd) (21). A total of 1.5 μg peptides were loaded onto a C18 trap column (100 μm I.D. × 2 cm) equilibrated with 12 μl solvent A (0.1% formic acid in water) and separated by an analytical column (150 μm I.D. × 15 cm, C18, 1.9 μm, 120 Å, Dr Maisch GmbH) with an EASY-nLC 1200 system (Thermo Fisher Scientific) using solvent A and solvent B (0.1% formic acid, 80% acetonitrile in water). The gradient was set as follows: 10% to 14% solvent B for 12 min, 14% to 26% solvent B for 45 min, 26% to 42% solvent B for 10 min, and 42% to 95% solvent B for 1 min. The peptides were then scanned on a Q Exactive HF mass spectrometer in DDA mode. Spectronaut pulsar × 12.0 (Biognosys) was performed for the construction of a spectral library by using BGS default parameter. The MS1 scan was set as 300 to 1400 m/z at a resolution of 60,000. The top twenty precursor ions were selected for MS2 by HCD fragmentation at a normalized collision energy of 28 with a resolution of 15,000. The automatic gain control was set to 3e6 for full MS1 and 5e4 for MS2, with maximum ion injection times of 80 and 100 ms, respectively. MS1/MS2 tolerance was fixed by using spectronaut default settings with software built-in automatic mass accuracy recognition and calibration algorithm.

Spectronaut pulsar × 12.0 (Biognosys) was used for quantification and DIA analysis. The MS1 was set at a resolution of 60,000 ranging from 350 to 1400 m/z. MS1/MS2 tolerance, retention time calibration, and window around the predicted time were both fixed by using spectronaut default settings with software built-in automatic mass accuracy recognition and calibration algorithm. None of the modification sites were used for analysis. The proteins, peptides, and peptide spectral matches levels were all set as 1% FDR. The DIA scans parameter was set with a resolution of 30,000; normalized collision energy: 28; automatic gain control target: 3e6; and maximal injection time: auto. Forty-five variable DIA windows were set for DIA acquisition. The sequential precursor isolation window setup was as follows: 374 to 412, 412 to 436.5, 436.5 to 457, 457 to 471.5, 471.5 to 483.5, 483.5 to 494.5, 494.5 to 507, 507 to 520.5, 520.5 to 533.5, 533.5 to 545, 545 to 554.5, 554.5 to 563.5, 563.5 to 573.5, 573.5 to 583.5, 583.5 to 593.5, 593.5 to 604, 604 to 615, 615 to 626, 626 to 636, 636 to 646, 646 to 657, 657 to 668.5, 668.5 to 680, 680 to 691, 691 to 702, 702 to 714, 714 to 726.5, 726.5 to 739.5, 739.5 to 753, 753 to 767, 767 to 781, 781 to 796, 796 to 812, 812 to 828.5, 828.5 to 846.5, 846.5 to 866, 866 to 887, 887 to 910, 910 to 935.5, 935.5 to 964, 964 to 998, 998 to 1040.5, 1040.5 to 1101, 1101 to 1269 m/z (supplemental Table S3). The DDA raw files were searched against the human FASTA database (downloaded on Jan 15, 2020, from UniProt) to generate a spectral library using the BGS factory setting. Carbamidomethyl (C) was set as fixed modification; oxidation (M) and acetyl (Protein N-term) were set as variable modifications. All results were filtered by a Q value cutoff of 0.01 [corresponding to a FDR of 1%]. The *p* value estimator was performed by Kernel Density Estimator. Proteins were removed from the mass spectrometry data if they were not detected in at least 80% of the samples. The identified proteins used in the spectral library construction and DIA analysis were shown in supplemental Tables S4 and S5, respectively.

#### Enzyme-Linked Immunosorbent Assay

The concentrations of PGAM1 and ENO1 were quantified by enzyme-linked immunosorbent assay with kits from mlbio following the manufacturer’s instructions. TIMP-1 concentration was determined with an ELISA kit from Sino Biological according to the manufacturer’s instructions. The samples were incubated on the plate for 2 h at room temperature and, once the stop solution was added, the optical density of each well was determined immediately using a microplate reader set to 450 nm by Varioskan Flash (Thermo Fisher Scientific).

#### Bioinformatics

The functional annotation of plasma proteins was executed using the PANTHER database (http://pantherdb.org/) (22). Since the data had a non-normal distribution, we analyzed the correlation between proteins and clinical variables (age, gender, Hans classification, IPI score) by Spearman correlation analysis using normalized data which was conducted by the R package “Hmisc” (https://CRAN.R-project.org/package=Hmisc), and correlation heat map clusters were calculated based on the Euclidian distance. Correlations were further assessed using a mixed effects model that adjusted for the four clinical factors and storage time using the “lmer” package (https://cran.r-project.org/web/packages/lme4/index.html). The normalized data was transformed to a Z-score (row-wise) prior to the hierarchical clustering analysis, and the heat map analysis was performed using R package “pheatmap” (https://cran.r-project.org/web/packages/pheatmap/). Prior to principal component analysis (PCA) analysis, the normalized data was scaled to 0 to 1 for further analysis by R package “factoextra” (https://cran.r-project.org/web/packages/factoextra/index.html). Differentially expressed proteins between DLBCL patients and HCs were subjected to GO and KEGG enrichment analyses using Omicsbean (http://www.omicsbean.cn/), Cytoscape, and ClueGO (23). The protein-protein network analysis was executed using STRING database (https://cn.string-db.org/). The hub proteins were identified by cytoHubba (24). Protein clustering and the Venn plot were calculated by the website Hiplot (https://hiplot.com.cn/). Protein lists generated from the four DLBCL subtypes were also subjected to enrichment analyses as described above.

#### Statistical Analysis

Prior to the statistical analysis, the missing values were imputed with the minimum value of each batch as previous described (17). To address batch-to-batch differences, protein data from antibody microarray and DIA-MS were normalized using the quantile method by the R statistical package limma (http://www.bioconductor.org/packages/release/bioc/html/limma.html).

The quartile-quartile plot and Shapiro–Wilk test showed that 94% of the proteins were not normally distributed (supplemental Fig. S2). To assess the reliability of sample size for the discovery phase, the power analyses were performed using Real Statistics Using Excel (https://www.real-statistics.com/non-parametric-tests/mann-whitney-test/mann-whitney-power/) to determine adjusted sample size and the pwr package (https://cran.r-project.org/web/packages/pwr/index.html). The majority (80%) of the proteins achieved a statistical power more than 0.8. All two-group comparisons were performed using the Wilcoxon rank-sum test (two-sided and unpaired) in this study. FDR adjusted by the Benjamini–Hochberg method was employed to estimate the probability of a false-positive finding with a threshold of 0.05 (25, 26).

We obtained 251 and 58 differentially expressed proteins (FDR < 0.05) from DIA-MS and antibody array, separately. Sixteen differentially expressed proteins were identified by both platforms (supplemental Table S6), in which we selected the data with the smaller *p* value for further statistical analysis as previously described (17). When comparing early-stage DLBCL patients and HCs, we obtained 197 and 21 differentially expressed proteins and six overlapped proteins were removed following the same strategy (supplemental Table S7).

For the NMF analysis, we calculated the fraction of total to remove the potential effects of dimensions. NMF is a general and robust method and is considered more precise than other unsupervised learning algorithms (27). To prove the robustness of the plasma proteomics subtypes revealed by the NMF analysis using the NMF package (https://cran.r-project.org/web/packages/NMF/index.html) in R, we also employed k-means and hierarchical clustering to cluster the DLBCL patients using the factoextra package (https://cran.r-project.org/web/packages/factoextra/index.html) in R https://www.R-project.org/. The kappa test was calculated by the irr package (https://CRAN.R-project.org/package=irr) to evaluate the similarity of the three algorithms.

Given a factorization rank k (where k represents the number of clusters), the NMF algorithm factorizes a non-negative target matrix “X” of dimension “n × p” into two non-negative matrices, “W” and “H,” where X≈WH. Matrix “W” is an n×k matrix representing the weights of samples (1 to n) in each cluster (1 to k), whereas matrix “H” is a k × p matrix representing the contribution of features (1 to p) in each cluster (1 to k). Using the method proposed by Kim *et al.* (28), matrix “W,” which contains the weights of each feature within a certain cluster, was used to derive a list of representative features separating the clusters. The resulting matrix was then subjected to NMF analysis using the NMF R package, which uses the “brunet” algorithm (29), and the stability of the clusters obtained from NMF was measured using the method proposed by Brunet *et al.* (27).

To determine the optimal factorization rank k (number of clusters) for the omic data matrix, a range of clusters between k = 2 and 6 was tested. For each k, we factorized matrix “V” using 50 iterations with random initializations of matrices “W” and “H.” By computing the cophenetic coefficient, the optimal k was determined to be k = 4 because it maximized the coefficient score. A consensus matrix with k = 4 appeared to have the clearest separation between clusters with the maximum cophenetic coefficient. To achieve robust factorization of the omics data from matrix “V,” we repeated the NMF analysis using 200 iterations with random initializations of matrices “W” and “H” and performed sample partitioning into clusters as described above.

To perform the survival analysis, OS was calculated from the start of the initial treatment until the time of death of any cause or until the last follow-up time point. Progression-free survival (PFS) was defined from the first day of treatment to the time of disease progression, recurrence, or death due to any cause. All OS and PFS calculations used the Kaplan–Meier method (log-rank test), which is a nonparametric method used for the survival analysis. The cut-off value of each candidate prognostic biomarker was determined by the R package ‘survminer,’ which was utilized to divide the cohort into two groups (high or low) for Kaplan–Meier analysis in validation cohorts. The hazard ratio was calculated from Cox proportional hazards regression analysis. Molecular features analyzed with a *p* value <0.05 using Cox regression univariate analysis were considered as significant and were included in the Cox regression multivariate analysis. The R package “survival” (https://cran.r-project.org/web/packages/survival/index.html) was used for the statistical tests of survival. The receiver operating characteristic curve analysis was drawn by R package “pROC” (https://cran.r-project.org/web/packages/pROC/index.html).

## Results

### Analysis of the DLBCL Plasma Proteome Using In-Depth Proteomics

A schematic illustration of this study is shown in Figure 1*A*. We enrolled 147 DLBCL patients and 79 HCs in the discovery cohort (Table 1). One hundred twenty-nine were treated with R-CHOP or R-CHOP–like chemotherapy regimens, one patient was treated with hyper-CVAD (hyper-fractionated cyclophosphamide, vincristine, doxorubicin, and dexamethasone), and the remaining patients were treated with CHOP (cyclophosphamide, doxorubicin, vincristine, and prednisone) or CHOP-like regimens (Fig. 1*B*).

**Fig. 2:**
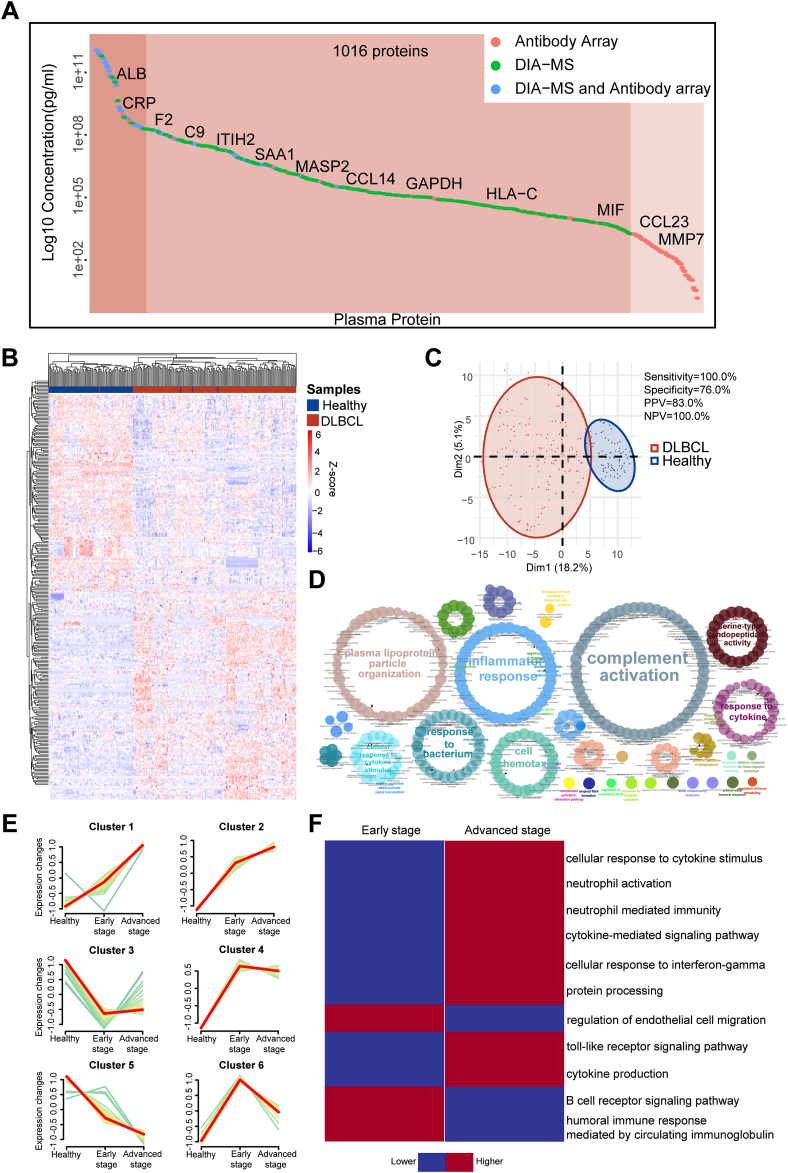
**Proteomic landscape of the plasma proteome in DLBCL patients.***A*, distribution of protein concentrations in plasma detected by in-depth proteomics was ordered according to the reference concentrations in the human plasma proteome database (http://www.plasmaproteomedatabase.org/). *B*, heat map of 293 differentially expressed proteins between DLBCL patients (n = 147) and HCs (n = 79). The signal densities of proteins were Z-scored across individuals. *C*, PCA plot of the 226 samples in the discovery cohort, demonstrating distinct protein profiles between DLBCL patients (n = 147) and HCs (n = 79). *D*, significantly enriched GO biological processes (*p* < 0.05) of the 293 differentially expressed proteins between DLBCL patients and HCs in the discovery cohort. *E*, six clusters representing different protein regulation trends from HCs to advanced stage DLBCL. *F*, significantly enriched biological processes (*p* < 0.05) of clusters 1, 2, and 5 from (*E*). DLBCL, diffuse large B-cell lymphoma; GO, gene ontology; HC, healthy control; NPV, negative predictive value; PCA, principal component analysis; PPV, positive predictive value.

The in-depth measurement of the plasma proteome was achieved by combining data from DIA-MS and customizable antibody microarray technologies due to their complementary detection: DIA-MS detects high- and middle-abundance proteins, whereas antibody microarrays can detect low-abundance proteins (17, 19, 30). A total of 1016 proteins were detected by DIA-MS and antibody microarray with 879 nonredundant proteins spanning ∼11 orders of magnitude based on information obtained from the human plasma proteome database (http://www.plasmaproteomedatabase.org/) (Fig. 2*A*) (31). We performed a Venn diagram analysis, which indicated that 140 proteins were detected with both DIA-MS and antibody array (supplemental Fig. S3). Functional annotation analyses revealed that plasma proteins detected by DIA-MS and the antibody microarray were enriched in the classes of defense/immunity and intercellular signaling, respectively (supplemental Fig. S4). The reproducibility of DIA-MS and antibody microarrays measurements was assessed using 20 replicates of HeLa cell lysates and four replicates of quality control plasma samples from HCs, respectively, with correlation coefficients of 0.9 to 1.0 (supplemental Fig. S5, *A* and *B*), demonstrating that our in-depth proteomics platform is reproducible.

In addition, we tested the influence of pre-analytical variables on the proteomics results. PCA of sample storage time indicated that the sampling year did not affect the data (supplemental Fig. S6, *A*–*C*), which is in accordance with a previous study of serum proteins using LC MS and Luminex bead-based immunoassays (32). Moreover, the storage temperature (−80 °C) and sample preprocessing time (<12 h) were consistent across all samples. Notably, according to previous reports, preprocessing times within 12 h have a minimal effect on serum or plasma proteins (18, 32).

### Alterations in the Plasma Proteome During DLBCL Pathogenesis

Using hierarchical clustering analysis (Fig. 2*B*) and PCA (Fig. 2*C*), two distinct clusters were made using 293 differentially expressed proteins (FDR < 0.05) identified with the Wilcoxon rank-sum test, with each cluster primarily DLBCL patients or HCs (supplemental Table S8). Ceruloplasmin (CP), a liver-secreted protein, drove the largest difference in the PCA plot, followed by the coagulation proteins and transport proteins, such as complement component C9 (C9), plasma protease C1 inhibitor (SERPING1), asparagine-tRNA ligase (NARS1), and transthyretin (supplemental Fig. S7).

Among the 293 proteins, the expression levels of 147 proteins were higher [Fold change (FC) ≥ 1)] and 146 proteins were lower (FC < 1) in DLBCL patients than in HCs (supplemental Fig. S8*A*). The three most increased proteins [(progranulin (GRN), vesicular integral-membrane protein VIP36 (LMAN2), and C-C motif chemokine 18 (CCL18)] are closely related to cell proliferation, glycoproteins transport, and B-cell migration, respectively (supplemental Fig. S8*B*). Some plasma functional proteins like serum albumin (ALB), complement C1r subcomponent (C1R), and complement C1q subcomponent subunit A (C1QA) (supplemental Fig. S8*C*) and proteins involved in the inflammatory response [*e.g.*, serum amyloid A-1 protein (SAA1), TIMP-1, CRP] (supplemental Fig. S8*C*) were also significantly increased.

GO enrichment analysis revealed that the 293 differentially expressed proteins were enriched in complement activation, inflammation response, plasma lipoprotein particle organization, cell chemotaxis, and response to cytokine (Fig. 2*D*). KEGG analysis indicated that upregulated proteins in DLBCL were enriched in complement and coagulation cascades, extracellular matrix-receptor interactions, the HIF-1 signaling pathway, and NF-kappa B signaling pathways (supplemental Fig. S9). However, the downregulated proteins in DLBCL were enriched in metabolism-associated pathways, including the PPAR signaling pathway and cholesterol metabolism (supplemental Fig. S9).

To elucidate the relationship between the plasma proteome and DLBCL progression, the DLBCL patients were separated into early (Ann Arbor stages I and II) or advanced stage (Ann Arbor stages III and IV) groups. Two hundred twelve proteins were differentially expressed (FDR < 0.05) in early-stage DLBCL patients compared to HCs. Using Hiplot (https://hiplot.com.cn/), these proteins represented six clusters with different protein profiles (Fig. 2*E* and supplemental Table S9). The expression of plasma proteins in clusters 4 and 6 was higher in early-stage DLBCL than in advanced-stage DLBCL. Cluster 3 proteins had the lowest expression in early-stage DLBCL than HCs and advanced-stage DLBCL.

The protein expression in clusters 1 and 2 was lowest in the HCs and then continually increased from early to advanced DLBCL, while the exact opposite was observed in cluster 5. The enriched biological processes of patient groups within clusters 1, 2, and cluster 5 were analyzed. Interestingly, upregulated proteins in early-stage DLBCL patients were enriched in humoral immunity processes, including B-cell receptor signaling pathway and humoral immune response mediated by circulating immunoglobulin (Fig. 2*F*). However, upregulated proteins in advanced-stage DLBCL patients were enriched in inflammation and cellular immunity processes, including cellular response to cytokine stimulus, cytokine-mediated signaling pathway, cytokine production, neutrophil activation, neutrophil-mediated immunity, cellular response to interferon-gamma, and toll-like receptor signaling pathway, etc. (Fig. 2*F*). These results reveal that different signaling pathways might be involved during the progression of DLBCL (33, 34, 35).

### DLBCL Subtyping Using In-Depth Plasma Proteomics

**Fig. 3:**
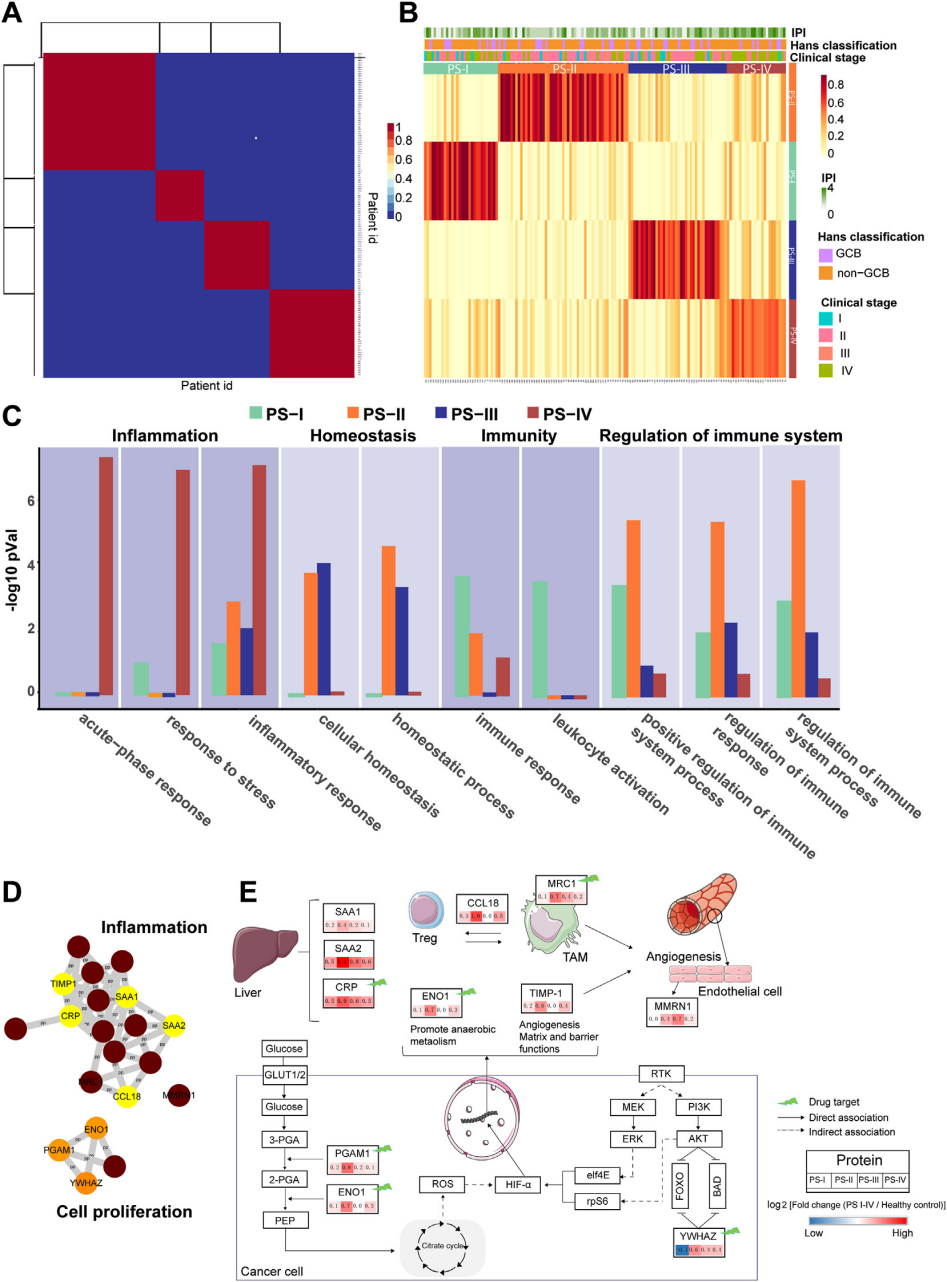
**Signature proteins and pathways in four DLBCL subtypes.***A*, heat map of the NMF consensus matrix. The x and y axes represent patients. *B*, NMF analysis of proteomic profiling identified four proteomic subtypes (DLBCL samples, n = 147): PS-I (*green*, n = 53), PS-II (*orange*, n = 40), PS-III (*blue*, n = 30), and PS-IV (*red*, n = 24). The associations of proteomic subtypes with clinical characteristics (IPI, Hans classification, and clinical stage) are annotated in the *middle* panel. *C*, representative biological process (*p* < 0.05) of the four subtypes. *D*, protein-protein interaction network of PS-IV proteins identified by STRING analysis and manual curation. The enrichment analysis was executed using STRING database in Cytoscape (version 3.7.2). *E*, cellular localization and biological signaling pathways of signature proteins representing the PS-IV subtype. The variations of signature proteins among four subtypes were defined as log_2_ (ratio of average protein abundance in each proteomic subtype *versus* HCs). *Red* and *blue* represent upregulated and downregulated proteins, respectively. The lightning symbols in *green* highlight proteins targeted by available drugs (approved, experimental, or investigational drugs). *Straight lines*, direct interaction; *dotted lines*, indirect interaction; TAM, tumor-associated macrophage; Treg, regulatory T cells. DLBCL, diffuse large B-cell lymphoma; HC, healthy control; IPI, international prognostic index; NMF, non-negative matrix fractionization; PS-IV, proteomics subtype IV.

Using NMF-based unsupervised clustering (Fig. 3, *A* and *B*) (27, 36), the DLBCL patients were clustered into two to six clusters, in which the optimal classification was k = 4 using cophenetic correlation coefficient and average silhouette width algorithms (supplemental Fig. S10*A*). Moreover, the rank of 4 generated the most stable classification in which the majority of the matrices correlation equals 0 or 1 (supplemental Fig. S10*B*).

Furthermore, we employed different unsupervised clustering methods, k-means and hierarchical clustering, to verify the clusters revealed by NMF. Like NMF, the DLBCL patients were consistently classified into four subtypes by k-means and hierarchical clustering (supplemental Fig. S11, *A* and *C*). Next, the kappa test was performed to determine how the proteomics classifications identified by these three clustering methods (NMF, k-means, hierarchical) correlated with each other. The kappa coefficient of k-means and NMF was 0.87, and the kappa coefficient of hierarchical clustering and NMF was 0.78 (supplemental Fig. S11*D*). These data indicate that different clustering methods similarly classified the four DLBCL subtypes, thus demonstrating the robustness of DLBCL subtyping *via* proteomics classification.

Twenty-nine proteins were metaproteins with same or similar expression patterns identified by NMF algorithm of the four proteomic subtypes (PS-I-IV) (supplemental Fig. S12). The tissue specificity and subcellular location of metaproteins were shown in supplemental Table S10. Notably, functional analysis revealed that the PS-I subtype contains proteins that are mainly enriched in immunity and regulation of immune system pathways, while the PS-II and PS-III subtypes contain proteins mainly enriched in the regulation of immune system and homeostasis pathways (Fig. 3*C*). Unlike the other proteomic subtypes, the PS-IV subtype contains ten proteins that are enriched in inflammatory and cell proliferation pathways, including those involved in the acute phase response, inflammatory response, and response to stress (Fig. 3*C*). STRING analyses indicate that nine proteins [TIMP-1, alpha-enolase (ENO1), CCL18, CRP, macrophage mannose receptor 1 (MRC1), phosphoglycerate mutase 1 (PGAM1), SAA1, serum amyloid A-2 protein (SAA2), 14-3-3 protein zeta/delta (YWHAZ)] that are expressed in liver tissue (supplemental Fig. S13) are highly associated with each other through direct and indirect interactions. Five (TIMP-1, CRP, MRC1, SAA1, SAA2) of these nine proteins are involved in the inflammatory process, while three proteins (PGAM1, ENO1, YWHAZ) are associated with cell proliferation (Fig. 3*D*) (37).

TIMP-1 is a metalloproteinase inhibitor of metalloproteinases with antiproteolytic and proinflammatory cytokine activity that can regulate inflammation, cell differentiation, and migration (38). It also promotes angiogenesis following the infiltration of tumor-associated macrophages (39). SAA1, SAA2, and CRP participate in the acute phase response (40). The upregulation of CRP is associated with a poor prognosis in follicular lymphoma patients undergoing rituximab-containing chemotherapy (40) and may indicate immunochemotherapy-related interstitial lung disease in B-cell lymphoma (41). CCL18 is mainly secreted by macrophagocytes, monocytes, and dendritic cells and may have functional roles in humoral and cell-mediated immunity responses. High expression of serological CCL18 is associated with significantly poor prognosis in patients of cutaneous T-cell lymphomas (42).

PGAM1 and ENO1 are important glycolytic enzymes that catalyze the conversion of 3-phosphoglycerate to phosphoenolpyruvate and coordinate glycolysis and biosynthesis that are important in cancer progression. PGAM1 and ENO1 are overexpressed in different cancers, while their inhibition may result in decreased tumor growth and metastasis (43, 44). Moreover, ENO1 and TIMP-1 supply tumors with energy for proliferation *via* the HIF-1α signaling pathway (45, 46). YWHAZ is an adapter protein implicated in a large spectrum of signaling pathways (*i.e.*, PI3K-AKT) (47), and it is upregulated in DLBCL patients with the activated B-cell–like subtype (48) (Fig. 3*E*).

### DLBCL Prognosis Based on Proteomic Subtypes

**Fig. 4:**
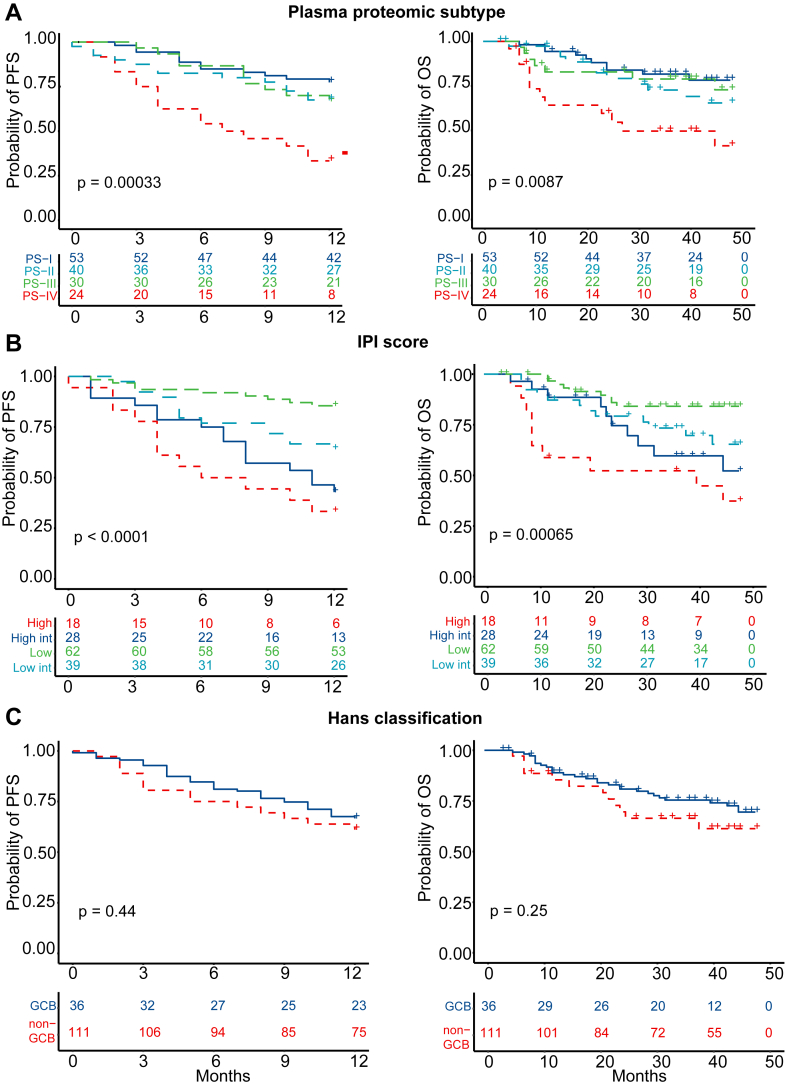
**Association of DLBCL proteomic subtypes with progression-free survival and overall survival.***A*, Kaplan–Meier models of 1-year PFS and 4-year OS according to DLBCL plasma proteomic subtypes. *p* value was calculated from log-rank test. *B*, Kaplan–Meier models of 1-year PFS and 4-year OS according to IPI score. *p* value was calculated from log-rank test. *C*, Kaplan–Meier models of 1-year PFS and 4-year OS according to Hans classification. *p* value was calculated from log-rank test. DLBCL, diffuse large B-cell lymphoma; High int, high intermediate; IPI, international prognostic index; low int, low intermediate; OS, overall survival; PFS, progression-free survival.

Regardless of the treatment regimens, the relationship between proteomic subtypes and the PFS and OS in DLBCL patients was ascertained (Fig. 4*A*). Patients with PS-I-III subtypes had more favorable outcomes, whereas those with the PS-IV subtype had unfavorable outcomes (Fig. 4*A* and Table 2). More specifically, the predicted 1-year PFS rates for the PS-I-IV subtypes were 77.4%, 67.5%, 66.7%, and 33.3%, respectively, while the predicted 4-year OS rates for the PS-I-IV subtypes were 78.2%, 65.4%, 72.8%, and 39.5%, respectively (Fig. 4*A* and Table 2). Next, the DLBCL patients were separated into four groups using IPI classification, with predicted 1-year PFS rates of 85.5%, 64.1%, 42.9%, and 33.3%, respectively, and predicted 4-year OS rates of 84.1%, 65.4%, 52.2%, and 37.3%, respectively (Fig. 4*B* and Table 2). The significant prognostic stratification based on the IPI score suggests that the sample cohort is heterogeneous. However, no statistical significance between GCB and non-GCB patient groups was found using the Hans classification system (Fig. 4*C*).

**Table 2:** One-year PFS and 4-year OS estimates for risk groups defined by three subtyping methods

Model	1-year PFS estimate (95%CI)	4-year OS estimate (95% CI)
Hans classification		
Non-GCB	66.7% (58.5%–76.0%)	69.5% (60.6%–79.8%)
GCB	61.1% (47.1%–79.3%)	61.3% (45.8%–82.0%)
IPI score		
Low (0–1)	85.5% (77.2%–94.7%)	84.1% (75.1%–94.2%)
Low intermediate (2)	64.1% (50.7%–94.7%)	65.4% (50.8%–84.1%)
High intermediate (3)	42.9% (27.9%–65.7%)	52.2% (33.8%–80.7%)
High (4–5)	33.3% (17.3%–64.1%)	37.3% (19.4%–72.0%)
Plasma proteomic subtype		
PS-I	77.4% (66.9%–89.5%)	78.2% (66.8%–91.6%)
PS-II	67.5% (54.4%–83.7%)	65.4% (50.7%–84.5%)
PS-III	66.7% (51.7%–85.9%)	72.8% (56.8%–93.3%)
PS-IV	33.3% (18.9%–58.7%)	39.5% (22.5%–69.4%)

**Fig. 5:**
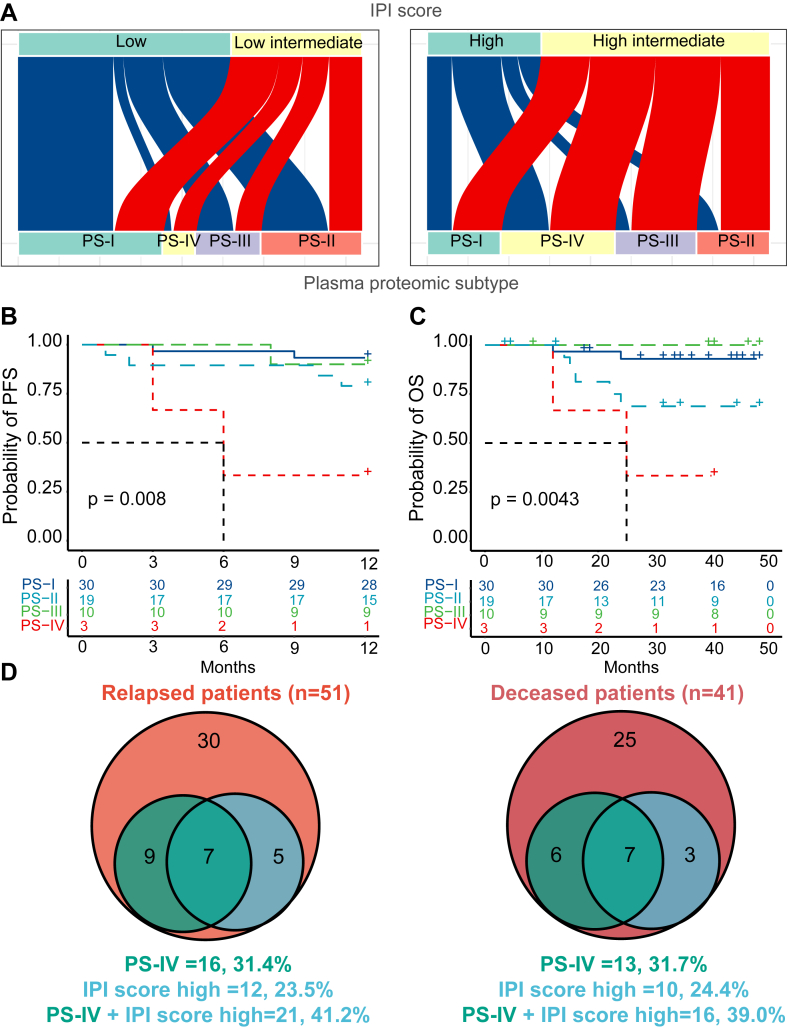
**The association of four proteomic subtypes with IPI score.***A*, the distribution of plasma proteomic subtypes within IPI subgroups. *B*, one-year PFS of four proteomic subtypes among low-risk IPIs. *p* value was calculated from log-rank test. *C*, four-year OS of four proteomic subtypes among low-risk IPIs. *p* value was calculated from log-rank test. *D*, a comparison of relapsed (1 year) and deceased (4 years) DLBCL patients following initial diagnosis based on plasma proteomic subtypes, IPI score, or both. DLBCL, diffuse large B-cell lymphoma; IPI, international prognostic index; OS, overall survival; PFS, progression-free survival.

The association between proteomic subtypes and the IPI score classification system was compared. The PS-I and PS-II subtypes were primarily classified as low-risk IPI. The PS-IV subtype included mostly high intermediate or high-risk IPI groups, whereas the PS-III subtype included all four IPI subgroups (Fig. 5*A*). Among low-risk IPIs, patients with the PS-IV subtype had significantly inferior 1-year PFS rates than patients with PS-I and PS-III subtypes (*p* = 0.008) (Fig. 5*B*). Patients with either PS-II or PS-IV subtypes had significantly inferior 4-year OS rates than PS-I–subtyped patients (Fig. 5*C*). Notably, we found that predicting DLBCL patients’ relapse and death was significantly improved by combining the proteomic subtype with IPI score (41.2% and 39.0%, respectively) than relying on the proteomic subtype (31.4% and 31.7%, respectively) or IPI score (23.5% and 24.4%, respectively) alone (Fig. 5*D*).

The association between proteomic subtypes and the Hans classification was also determined. Of the 147 cases, 36 (24.5%) were considered GCB, and 111(75.5%) were considered non-GCB by the Hans classification system (Table 1). The 1-year PFS and 4-year OS rates were not significantly different between the two groups. All four proteomic subtypes included GCB and non-GCB cases (supplemental Fig. S14*A*). Of the GCB cases, PS-I accounted for 38.89%, PS-II for 19.44%, PS-III for 22.22%, and PS-IV for 19.44% (supplemental Fig. S14*B*). Of the non-GCB cases, 35.13% were classified as PS-I, 29.73% were PS-II, 19.82% were PS-III, and 15.32% were PS-IV (supplemental Fig. S14*B*). Within non-GCB DLBCL patients, the 1-year PFS rates (*p* = 0.011) and 4-year OS rates (*p* = 0.02) of the four proteomic subtypes were distinct from each other (supplemental Fig. S14, *C* and *D*). The PS-IV subtype was associated with a significantly inferior prognosis than the PS-I-III subtypes (supplemental Fig. S14, *C* and *D*).

These results indicate that the prognostic value of proteomic subtypes is independent of the IPI score and Hans classification system. However, using the proteomic subtypes, IPI score, and Hans classification system together may help identify DLBCL patients with poor prognosis. As such, these patients could be treated with alternative options that may be more effective for them.

Next, we conducted a multivariate analysis to test the influence of inflammation (white blood cell, lymphoma count, neutrophil, CRP), renal (creatinine, blood urea nitrogen), and liver (aspartate aminotransferase, alanine aminotransferase) functions on the proteomic subtypes in DLBCL prognosis using appropriate clinical variables (supplemental Fig. S15). The PS-IV was still significantly associated with a poor prognosis, with a hazard ratio higher than 2 after adjusting for the clinical variables associated with inflammation, renal, and liver functions.

Lastly, we investigated whether proteomics subtyping was associated with clinical phenotypes. Patients with the worst prognosis that were classified as PS-IV with proteomics analysis had advanced stage (66.67%) as well as the the highest number of extranodal involvement and abnormal lactate dehydrogenase expression (supplemental Fig. S16). Interestingly, the ability to accurately predict patient prognosis could not be performed based on the clinical data alone (sex, extranodal involvement, bone marrow involvement, B symptom, lactate dehydrogenase above normal, Ann Arbor Stage, age) (supplemental Fig. S17, *A*–*D*). These results demonstrate the usefulness of proteomics subtyping in helping to predict the prognosis of DLBCL patients.

### Identification of Protein Biomarkers of Poor Prognosis

**Fig. 6:**
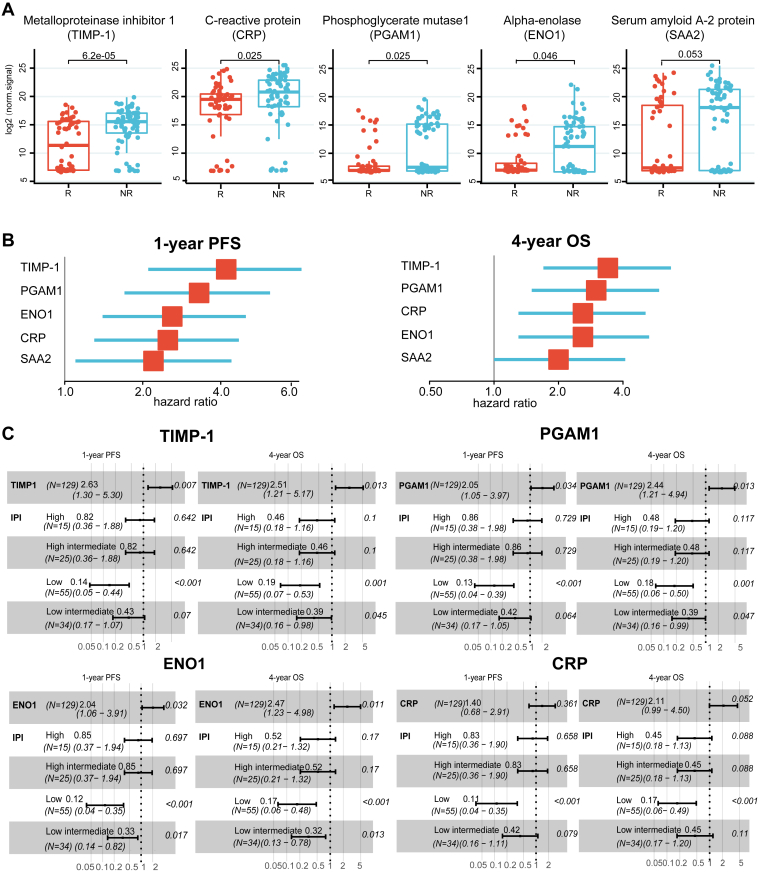
**Selection of protein biomarkers in the discovery cohort.***A*, boxplots showing significant differences (*p* < 0.05) in signature proteins between R (n = 56) and NR (n = 73) patients by Wilcoxon rank-sum test (two-sided, nonpaired) in the discovery cohort. The *central line* is the median, bounds of *box* represent the first and third quartiles, and the *upper* and *lower* whiskers represent the considering outliers within the 1.5× interquartile range (IQR) where IQR equals the third quantile minus the first quantile. *B*, forest plot demonstrating the association between five signature proteins and PFS and OS in the R-CHOP/R-CHOP-like treatment cohort by univariate Cox regression analysis. The *square points* in *red* represent the hazard ratio of each protein; the *blue lines* indicate the lower or upper 95% confidence interval. *C*, the association of four biomarker candidates with PFS and OS when adjusted by the IPI score using multivariate Cox analysis. IPI, international prognostic index; NR, non-responder; OS, overall survival; PFS, progression-free survival; R, responder.

Since the PS-IV subtype is associated with a poor prognosis in DLBCL patients, further evaluation of proteins within this subtype was performed to identify specific protein biomarkers with the highest predictive power. To address this concern, the expression of PS-IV subtype proteins between R and NR patients to R-CHOP/R-CHOP–like treatment in the discovery cohort was first compared. Four dysregulated proteins were identified, including TIMP-1, CRP, PGAM1, and ENO1 (Wilcoxon rank-sum test, *p* < 0.05) (Fig. 6*A*). Although marginally significant (*p* = 0.053) in the discovery cohort, SAA2 was selected due to its function as an indicator of special humoral immune responses in B-cell lymphoma induced by germinal center–associated lymphoma (HGAL) genes (49).

Next, the individual capability of each biomarker in predicting clinical outcomes was evaluated in the discovery cohort using the univariate Cox regression analysis. All five proteins (TIMP-1, CRP, PGAM1, ENO1, SAA2) were significantly associated with 1-year PFS and 4-year OS, with TIMP-1 having the highest hazard ratio (Fig. 6*B*). The multivariable Cox analyses of time-to-event endpoints further indicate that high levels of three proteins (TIMP-1, PGAM1, ENO1), independent of the IPI score, were significantly associated with worse 1-year PFS and 4-year OS (*p* < 0.05) (Fig. 6*C* and supplemental Table S11).

### Validation of Biomarkers in Two Independent Cohorts

**Fig. 7:**
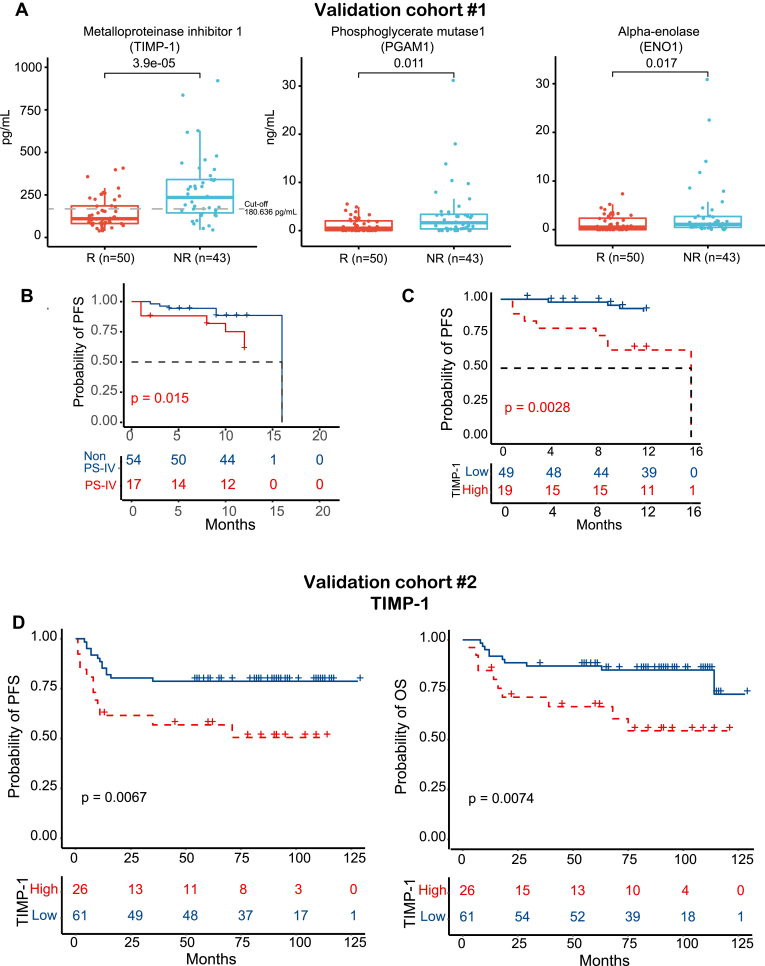
**Validation of DLBCL prognostic biomarkers in patients treated with R-CHOP.***A*, boxplots of TIMP-1, PGAM1, and ENO1 between R (n = 50) and NR (n = 43) patient groups in validation cohort #1. The three proteins were significantly expressed (*p* < 0.05) between R (n = 50) and NR (n = 43) groups using Wilcoxon rank-sum test (two-sided, nonpaired). The *central line* is the median, bounds of *box* represent the first and third quartiles, and the *upper* and *lower* whiskers represent the considering outliers within the 1.5× interquartile range (IQR) where IQR equals the third quantile minus the first quantile. *B*, Kaplan–Meier models of PFS between patient groups with PS-IV and non-PS-IV subtype in validation cohort #1. *p* value was calculated from log-rank test. *C*, Kaplan–Meier models of 1-year PFS of TIMP-1. *p* value was calculated from log-rank test. *D*, Kaplan–Meier models of PFS and OS between patient groups with high (“TIMP-1 high”) and low (“TIMP-1 low”) expression levels of TIMP-1 in validation cohort #2. *p* value was calculated from log-rank test. DLBCL, diffuse large B-cell lymphoma; NR, non-responder; OS, overall survival; PFS, progression-free survival; PS-IV, proteomics subtype IV; R, responder.

Using validation cohort #1 comprised of 93 DLBCL patients treated with R-CHOP or R-CHOP–like regimens, we validated three biomarkers (TIMP-1, PGAM1, ENO1) using ELISA technology. The results indicated that the expression levels of three proteins were significantly elevated in NR patients (n = 43) than in R patients (n = 50) (*p* < 0.001, *p* = 0.011, *p* = 0.017, respectively) (Fig. 7*A*), demonstrating the capability of these biomarkers in predicting the response of R-CHOP treatment. We built a subtyping model based on the random forest algorithm to predict PS-IV based on TIMP-1, PGAM1, and ENO1. We divided the discovery cohort into a training (n = 103) and test dataset (n = 44), with validation cohort #1 as the external validation cohort (n = 71). The area under the curves of the subtyping model in training and test datasets could reach 0.83 and 0.80 for predicting the PS-IV subtype (supplemental Fig. S18*A*). In the external validation cohort, the predicted PS-IV subtype had significantly shorter PFS than the non-PS-IV subtype (Fig. 7*B*).

TIMP-1 expression was significantly associated with patient survival (*p* = 0.0028) *via* Kaplan–Meier analysis when the patients were divided into ‘TIMP-1 high’ and ‘TIMP-1 low’ groups by cut-off value 180.636 pg/ml (Fig. 7*C* and supplemental Table S12). No statistical difference in patient survival was identified for either PGAM1 or ENO1 (supplemental Fig. S18*B*). Meanwhile, the expression of TIMP-1 was consistently elevated in DLBCL patients than in HC (supplemental Fig. S18*C*). Using a second validation cohort #2 comprised of 87 DLBCL patients, a high level of TIMP-1 (cut-off value = 180.636 pg/ml) was also significantly associated with poor 1-year PFS (*p* = 0.0067) and 4-year OS (*p* = 0.0074) (Fig. 7*D* and supplemental Table S12). In addition, high levels of TIMP-1 mRNA were also associated with a poor prognosis in a Western cohort (GSE31312) from the Gene Expression Omnibus database (50, 51). These results indicate that TIMP-1 could serve as a prognostic biomarker of DLBCL patients in Asian and Western populations (supplemental Fig. S19).

We gathered post-treatment samples from 27 patients classified as NR. These samples included 17 patients who collected samples at progression and ten patients who were in remission at the fourth cycle of treatment. Our findings indicated that TIMP-1 levels were elevated during progression and reduced after remission (supplemental Fig. S20).

### TIMP-1 can Stratify Low-Risk IPIs and Increase the Predictive Sensitivity of the IPI Scoring System

**Fig. 8:**
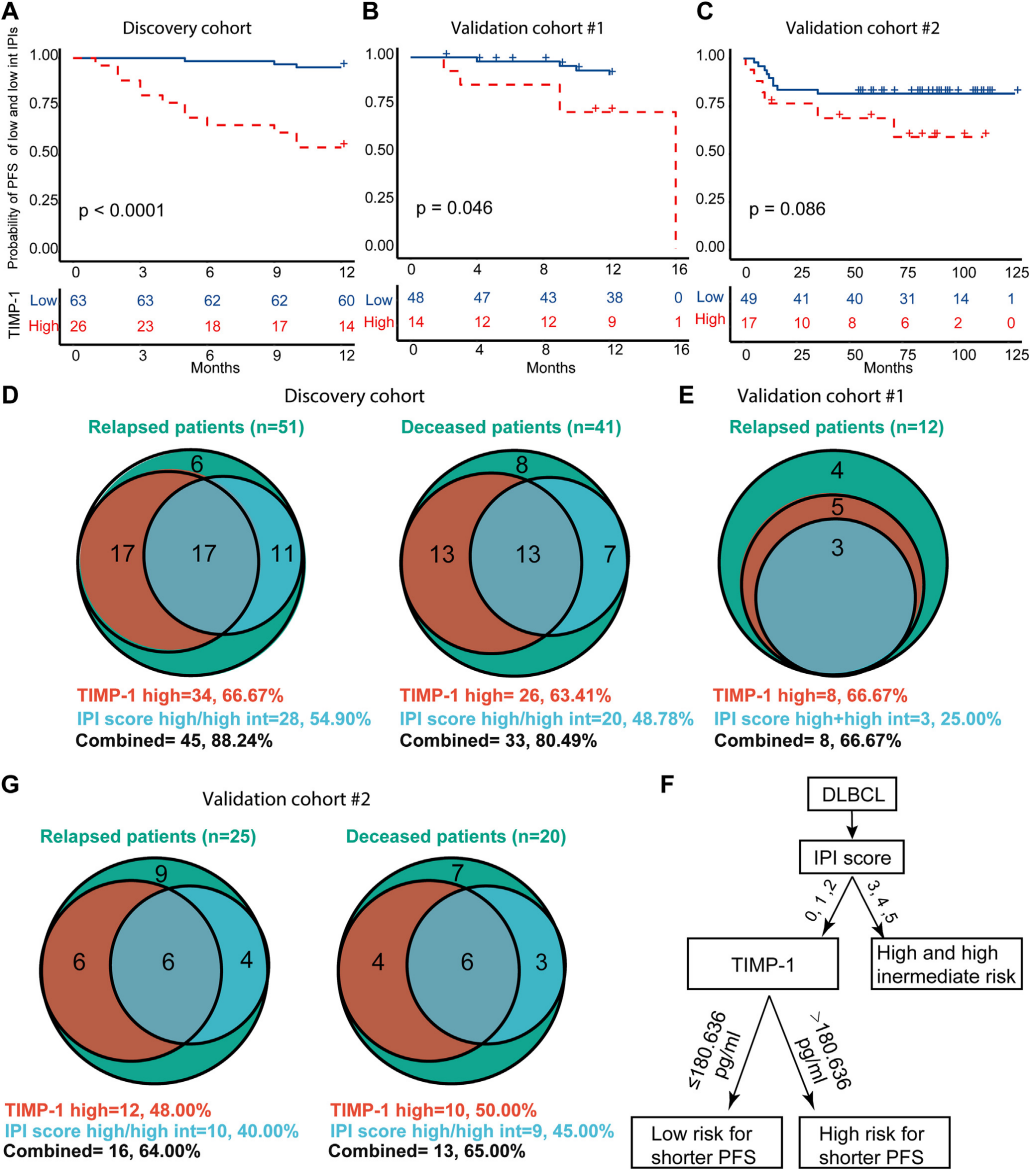
**TIMP-1 can stratify low-risk IPIs and increase the predictive sensitivity of the IPI scoring system.***A*–*C*, Kaplan–Meier models of 1-year PFS within low-risk IPIs (IPI=0, 1, 2) based on serological TIMP-1 expression levels in the discovery cohort, validation cohort #1, and validation cohort #2. *p* value was calculated from log-rank test. *D*, comparison of accuracy of using TIMP-1 expression levels and the IPI score in identifying relapsed and deceased patients within the discovery cohort. *E*, comparison of accuracy of using TIMP-1 expression levels and the IPI score in distinguishing relapsed patients in the validation cohort #1. *F*, comparison of accuracy of using TIMP-1 expression levels and the IPI score in distinguishing relapsed and deceased patients among validation cohort #2. *G*, the decision tree was constructed using TIMP-1 and IPI score. Using the decision tree, the patients can be classified as the high risk for shorter PFS by the high IPI score (3–5) and low IPI score (0–2) with TIMP-1 expression higher than the cut-off (180.636 pg/ml). IPI, international prognostic index; PFS, progression-free survival.

Some patients (15%) classified by the IPI scoring system as having low or low-intermediate risk still had poor outcomes (Fig. 5, *A*–*C*), thus it is of great significance to identify such patients. Of the 89 DLBCL patients with low or low-intermediate IPI scores in the discovery cohort treated with R-CHOP/R-CHOP–like therapy, 29.2% (26/89) of the patients had high TIMP-1 expression levels (above the third quartile) while 70.8% (63/89) had low TIMP-1 expression levels (below the third quartile). These two patient groups had significant differences in outcome (Fig. 8*A* and supplemental Fig. S21*A*). Similar results were obtained with validation cohorts #1 and #2, in which 22.5% (14/62) and 25.7% (17/66) of patients misclassified by IPI score were correctly identified by TIMP-1 expression level, respectively (Fig. 8, *B* and *C* and supplemental Fig. S21*A*). Meanwhile, early-stage DLBCL could also be classified by TIMP-1 into high- or low-risk groups with significantly distinct prognoses (supplemental Fig. S21*B*).

Additionally, we investigated whether the TIMP-1 and IPI score could predict which patients would relapse or not survive using three independent patient cohorts (*i.e.*, discovery, validation cohort #1, validation cohort #2). Notably, TIMP-1 expression level had higher accuracy than the IPI score in all three cohorts in predicting patients who will relapse (discovery: 66.7% *versus* 54.9%, validation cohort #1: 66.7% *versus* 25.0%, validation cohort #2: 48.0% *versus* 40.0%, respectively) or not survive (discovery: 63.4% *versus* 48.8%, validation cohort #2: 50.0% *versus* 45.0%, respectively) (Fig. 8, *D*–*F*). No survival data were available for validation cohort #1.

Combining TIMP-1 expression levels with the IPI score improved the predictive power, increasing the accuracy to 88.2%, 66.7%, and 64.0% for relapsed patients across the discovery cohort, validation cohort #1, and validation cohort #2, respectively (Fig. 8, *D*–*F*). These values represent a 24.00%-41.67% improvement compared to the IPI score alone. For decease prediction, the TIMP-1/IPI score combination reached 80.5% and 65.0% in the discovery cohort and validation cohort #2, respectively, which improved the predictive power of the IPI score alone by 20.00%-31.70% (Fig. 8, *D*–*F*). In order to apply TIMP-1 in clinics, we developed a decision tree model by combining TIMP-1 and IPI score for DLBCL risk stratification (Fig. 8*G*). Specifically, the patients can be classified as the high risk for shorter PFS by a high IPI score (3–5) or a low IPI score (0–2) with TIMP-1 expression higher than the cut-off value (180.636 pg/ml).

## Discussion

Knowledge about DLBCL pathogenesis and heterogeneity remains limited. However, understanding such information is important to improve patient care since 40% to 60% of DLBCL patients have unfavorable outcomes (*e.g.*, relapse, poor survival) after receiving traditional R-CHOP/R-CHOP–like therapy (34). While the availability of clinical biopsy samples is challenging, minimally invasive sample types like plasma or serum containing circulating proteins would be easier to obtain for studying the disease and identifying biomarkers for diagnosis and therapy (15, 52, 53, 54, 55).

In this work, a proteome-wide analyses of plasma in 147 DLBCL patients and 79 HCs was performed using DIA-MS and customizable antibody microarrays. The data revealed that the DLBCL is associated with potentially upregulated biological processes, such as acute phase response, humoral immune response, inflammation response, hemostasis, protein activation cascade, and blood coagulation in DLBCL patients (Figs. 1 and 2). While early-stage DLBCL patients might have upregulated humoral immunity, advanced-stage DCBCL patients may have increased inflammation and cellular immunity (Fig. 3, *A* and *B*).

Using in-depth plasma proteomics, the DLBCL patients were classified into four proteomic subtypes. Three subtypes (PS-I-III) were associated with a good prognosis and were associated with the regulation of the immune response and homeostasis. The PS-I, PS-II, and PS-III subtypes did not exhibit different significantly PFS and OS which may be caused that the three subtypes have similar biological functions in these patients that were determined by proteomics analysis. Specifically, the PS-I, PS-II, and PS-III subtypes contain proteins that are commonly enriched in the regulation of immune system pathways (Fig. 3*C*). The results are in accordance with transcriptomics data obtained in previously studies, which showed that patients with a good prognosis had an overexpression of genes in germinal center B cells in the GCB subtype (3, 6). Moreover, two genetic subtypes (clusters 1 and 4) with a good prognosis were identified. Differentially expressed transcripts (*e.g.*, CD70, BCL10, BCL-6, HLA-B) in cluster 1 are involved in the adaptive immune response, while gene transcripts differentially expressed in cluster 4 regulate lymphocyte and T cell differentiation, gene expression, and primary metabolic process (4). In another study, Wright *et al.* (8) classified DLBCL patients into seven genetic subtypes using the LymphGen algorithm, including MCD, BN2, N1, EZB/MYC+, EZB/MYC-, A53, and ST2. Interestingly, the ST2 subtype with the highest 5-year OS in three cohorts (National Cancer Institute, Harvard, BC Cancer Agency) had upregulated gene transcripts in GCB cells. The subtype PS-IV with a poor prognosis (Fig. 4*A*) is characterized by differentially expressed proteins involved in the inflammatory response, acute phase response, and response to stress; however, correlated genes were not found in genomic and transcriptomic studies (3, 4, 6, 8). Notably, the potential utility of proteomic subtypes in the prognosis of DLBCL patients was demonstrated when used in conjunction with the IPI and Hans classification systems (Figs. 4 and 5; supplemental Fig. S21). This study provides new proteomic insights into the molecular heterogeneity of DLBCL patients.

To demonstrate the translational potential of our proteomics results in the clinic, it is necessary to validate potential biomarkers in an independent cohort using a different technology, such as ELISA, that can quantify the biomarker in a large number of clinical samples easily (17, 30, 56). To address this concern, three differentially expressed proteins (TIMP-1, PGAM1, ENO1) between NR and R groups following R-CHOP/R-CHOP–like regimens were identified in the discovery cohort. These potential biomarkers could help predict the therapeutic response of R-CHOP/R-CHOP–like regimens in the clinic. We found proteins that differ hugely between DLBCL and HCs may not be suitable for prognostic prediction. For example, based on supplemental Fig. S6, we selected the top two proteins CP and C9 and we also included GRN which has the highest fold change when comparing DLBCL and HC. We found that C9 and GRN did not differ between R and NR. Though CP is differently expressed between R and NR, it did not show prognostic value for PFS and OS. Importantly, a high expression level of TIMP-1 in PS-IV was ranked as the top risk factor of a poor prognosis (*i.e.*, poor 1-year PFS and 4-year OS) (Fig. 6) for all three patient cohorts. Moreover, TIMP-1 can complement the IPI score classification system (Fig. 8), improving the accuracy of prediction of relapsed and deceased DLBCL patients compared to using the IPI score alone (Fig. 8, *D*–*F*).

TIMP-1 contributes to cancer progression and pathogenesis in a complex way due to its versatile impact on cellular functions stemming from its two-domain structure (57). Both TIMP-1 immunohistochemistry staining and serological TIMP-1 levels have prognostic values in lung, melanoma, breast, colon, and several other cancers (58, 59, 60, 61). While it is known that TIMP-1 promotes DLBCL progression by regulating cell migration and the Wnt signaling pathway (62), the prognostic value of TIMP-1 in DLBCL has not been fully studied, especially in patients treated with CHOP. This study investigated the prognostic value of TIMP-1 in DLBCL patients treated with R-CHOP. The roles of TIMP-1 in this disease will be conducted in future studies. Furthermore, the prognostic value of TIMP-1 expression levels in tissue is still controversial, with two studies concluding its relevance while another study did not. In this study, we demonstrate that serological TIMP-1 can be an independent biomarker of prognosis in patients receiving R-CHOP. In addition, measuring TIMP-1 in plasma can be easily performed using conventional immunoassays (*e.g.*, ELISA) that are highly sensitive, simple to perform, cost-effective, and can be easily automated in a clinical laboratory.

There are several limitations in this study. First, the number of clinical samples used in this study was limited, and the candidate biomarkers should be validated in a larger cohort in the future. Second, genomics and transcriptomics analyses were not performed in this study due to the unavailability of peripheral blood mononuclear cells of these cohorts. Third, although the TIMP-1 showed promising results in DLBCL prognosis (Figs. 7 and 8), however, the capability of TIMP-1 to serve as the diagnostic biomarker might be limited to discriminate the DLBCL patients from healthy control and other disease patients which remain to be investigated in the future (supplemental Fig. S18*A*). In addition, only patients being treated with R-CHOP/R-CHOP–like treatment regimens were studied. Thus, the prognostic value of TIMP-1 may not apply to other treatments. Finally, other health conditions that could affect the prognosis of DLBCL patients, such as the healthy history and Epstein-Barr virus infection, were not investigated in this study.

## Conclusion

In this work, we demonstrated the utility of plasma proteomics. In addition to showcasing DLBCL heterogeneity, plasma proteomics enabled the identification of circulating biomarkers that could be used for risk stratification in DLBCL patients receiving R-CHOP treatment. Our proteomics pipeline could serve as a paradigm for translational studies of different cancers in the future. Moreover, we identified TIMP-1 as a prognostic biomarker that could be used in conjunction with the current IPI scoring system to accurately identify patients who would benefit from R-CHOP treatment accurately.

## Data Availability

The datasets produced in this study are available in iProX: URL: https://www.iprox.cn/page/PSV023.html;?url=1686313360111EwR5; Password: X1WO.

## Supplemental Data

This article contains supplemental data.

## Conflict of interest

The authors declare no competing interests.
