# Supplementary material for: Proteomics Identifies Circulating TIMP-1 as a Prognostic Biomarker for Diffuse Large B-Cell Lymphoma
Source: Mol Cell Proteomics. 2023 Jul 26;22(9):100625. doi: 10.1016/j.mcpro.2023.100625 (PMC10470290; doi:10.1016/j.mcpro.2023.100625)
Supplement: Supplemental Information [file mmc1.docx]

**Appendix for**

**Proteomics identifies circulating TIMP-1 as a prognostic biomarker for diffuse large B-cell lymphoma**

Authors: Ning Lou^1†^, Guibin Wang^2†^, Yanrong Wang^3†^, Meng Xu^2^, Yu Zhou^3^, Qiaoyun Tan^3^, Qiaofeng Zhong^3^, Lei Zhang^3^, Xiaomei Zhang^2^, Shuxia Liu^1^, Rongrong Luo^1^, Shasha Wang^1^, Le Tang^3^, Jiarui Yao^3^, Zhishang Zhang^3^, Yuankai Shi^3*^, Xiaobo Yu^2*^, Xiaohong Han^4*^

Affiliations:

^1^Department of Clinical Laboratory, National Cancer Center/National Clinical Research Center for Cancer/Cancer Hospital, Chinese Academy of Medical Sciences & Peking Union Medical College, Beijing Key Laboratory of Clinical Study on Anticancer Molecular Targeted Drugs; No. 17 Panjiayuan Nanli, Chaoyang District, Beijing 100021, China.

^2^State Key Laboratory of Proteomics, Beijing Proteome Research Center, National Center for Protein Sciences-Beijing (PHOENIX Center), Beijing Institute of Lifeomics; Beijing 102206, China.

^3^Department of Medical Oncology, National Cancer Center/National Clinical Research Center for Cancer/Cancer Hospital, Chinese Academy of Medical Sciences & Peking Union Medical College, Beijing Key Laboratory of Clinical Study on Anticancer Molecular Targeted Drugs; No. 17 Panjiayuan Nanli, Chaoyang District, Beijing 100021, China.

^4^Clinical Pharmacology Research Center, Peking Union Medical College Hospital, State Key Laboratory of Complex Severe and Rare Diseases, NMPA Key Laboratory for Clinical Research and Evaluation of Drug, Beijing Key Laboratory of Clinical PK & PD Investigation for Innovative Drugs，Chinese Academy of Medical Sciences & Peking Union Medical College; No.1, Shuaifuyuan, Dongcheng District, Beijing 100730, China.

*Correspondence: hanxiaohong@pumch.cn; yuxiaobo@ncpsb.org.cn; syuankai@cicams.ac.cn

† These authors contributed equally to this work

**Appendix Figures**

| Appendix Figure S1 | Comparison of clinical markers of liver and renal function in DLBCL patients and HCs | Page 5 | |
| --- | --- | --- | --- |
| Appendix Figure S2 | Quantile-Quantile plot analysis of proteomics dataset | Page 6 | |
| Appendix Figure S3 | Venn diagram of proteins identified by antibody array and DIA-MS analyses | Page 7 | |
| Appendix Figure S4 | Enriched classes of differential plasma proteins in DLBCL patients compared to HCs | Page 8 |  |
| Appendix Figure S5 | Reproducibility of plasma proteome detection using DIA-MS and antibody arrays | Page 9 |  |
| Appendix Figure S6 | PCA plot of samples collected at different times | Page 10 |  |
| Appendix Figure S7 | Contribution of each variable to the separation of principal component (PC) 1 and PC2 in the PCA | Page 11 |  |
| Appendix Figure S8 | Identification of differentially-expressed proteins between DLBCL patients and HCs | Page 12 |  |
| Appendix Figure S9 | Enriched KEGG pathways of dysregulated proteins in the plasma of DLBCL patients compared to HCs | Page 13 |  |
| Appendix Figure S10 | NMF quality metrics | Page 14 |  |
| Appendix Figure S11 | DLBCL subtypes identified by k-means or hierarchical clustering methods using proteomic data | Page 15 |  |
| Appendix Figure S12 | Hierachical clustering analysis of signature proteins in the four proteomic subtypes | Page 16 |  |
| Appendix Figure S13 | Expression of PS-IV proteins in liver tissue | Page 17 |  |
| Appendix Figure S14 | Classifying DLBCL patients based on proteomic subtypes or the Hans classification system | Page 18 |  |
| Appendix Figure S15 | The prognostic value of plasma proteomic subtypes adjusted by inflammation, liver and kidney function using multivariate Cox analysis | Page 19 |  |
| Appendix Figure S16 | Association between plasma proteomic subtypes and the clinical variables | Page 20 |  |
| Appendix Figure S17 | DLBCL subtypes identified by clinical data with the NMF method | Page 21 |  |
| Appendix Figure S18 | The AUC of the subtyping model and the prognostic value of PGAM1 and ENO1 | Page 22 |  |
| Appendix Figure S19 | The prognostic value of TIMP-1 mRNA using the GSE31312 dataset | Page 23 |  |
| Appendix Figure S20 | Effect of R-CHOP or R-CHOP-like regimens on TIMP-1 level | Page 24 |  |
| Appendix Figure S21 | Stratification of 4-year OS in DLBCL patients with low-risk IPIs and early-stage based on the level of TIMP-1 in plasma | Page 25 |  |

**Appendix Tables**

| Appendix Table S1 | Summary of different DLBCL classifications obtained to date | Page 26 |
| --- | --- | --- |
| Appendix Table S2 | Clinical information of the patients from three cohorts |  |
| Appendix Table S3 | The parameters of forty-five DIA windows in DIA acquisition |  |
| Appendix Table S4 | The detailed protein information used in the spectral library construction |  |
| Appendix Table S5 | The detailed protein information used in DIA analysis |  |
| Appendix Table S6 | The list of proteins that differ significantly between DLBCL and HCs detected by both DIA-MS and antibody arrays | Page 27 |
| Appendix Table S7 | The list of proteins that differ significantly between early-stage DLBCL and HCs detected by both DIA-MS and antibody arrays | Page 28 |
| Appendix Table S8 | The full names and abbreviations of 293 differentially expressed proteins |  |
| Appendix Table S9 | List of plasma proteins with the normalized value in the six DLBCL clusters identified with proteomics | Page 29-33 |
| Appendix Table S10 | The tissue specificity and subcellular location of metaproteins |  |
| Appendix Table S11 | The detection of ENO1, PGAM1 and TIMP-1 by DIA-MS in patients from discovery cohort |  |
| Appendix Table S12 | The detection of TIMP-1 by ELISA in validation #1 and #2 cohorts patients |  |

**Appendix Figures**


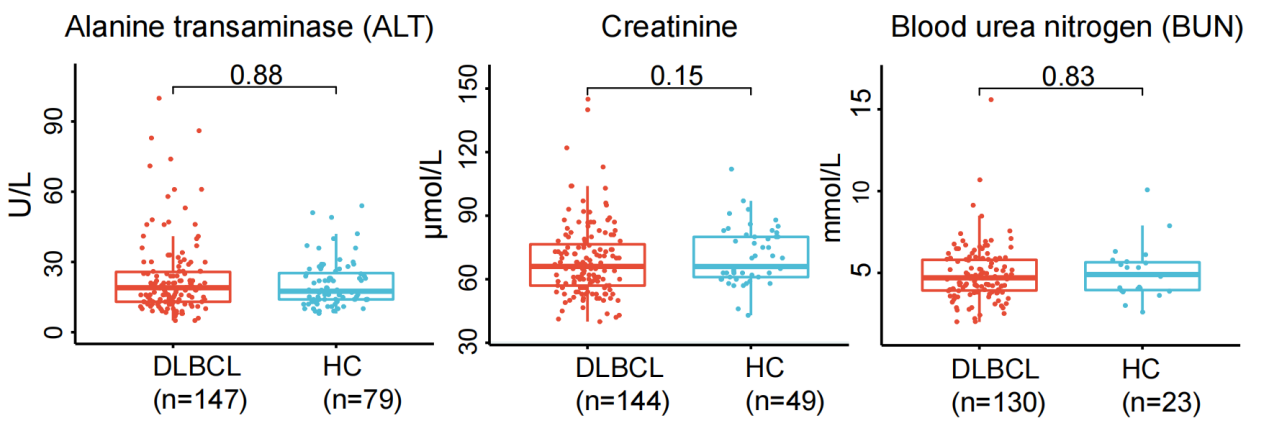


**Appendix Figure S1. Comparison of clinical markers of liver and renal function in DLBCL patients and HCs.**


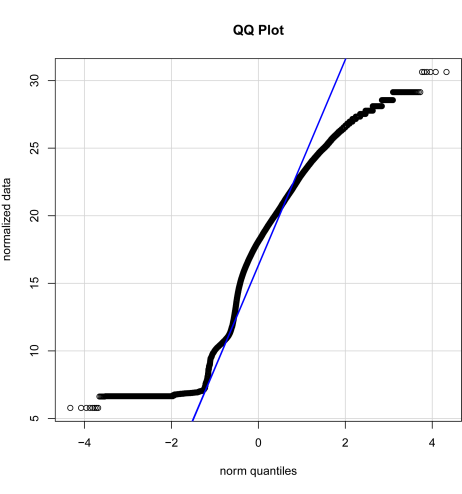


**Appendix Figure S2. Quantile-Quantile plot analysis of proteomics dataset.**

**
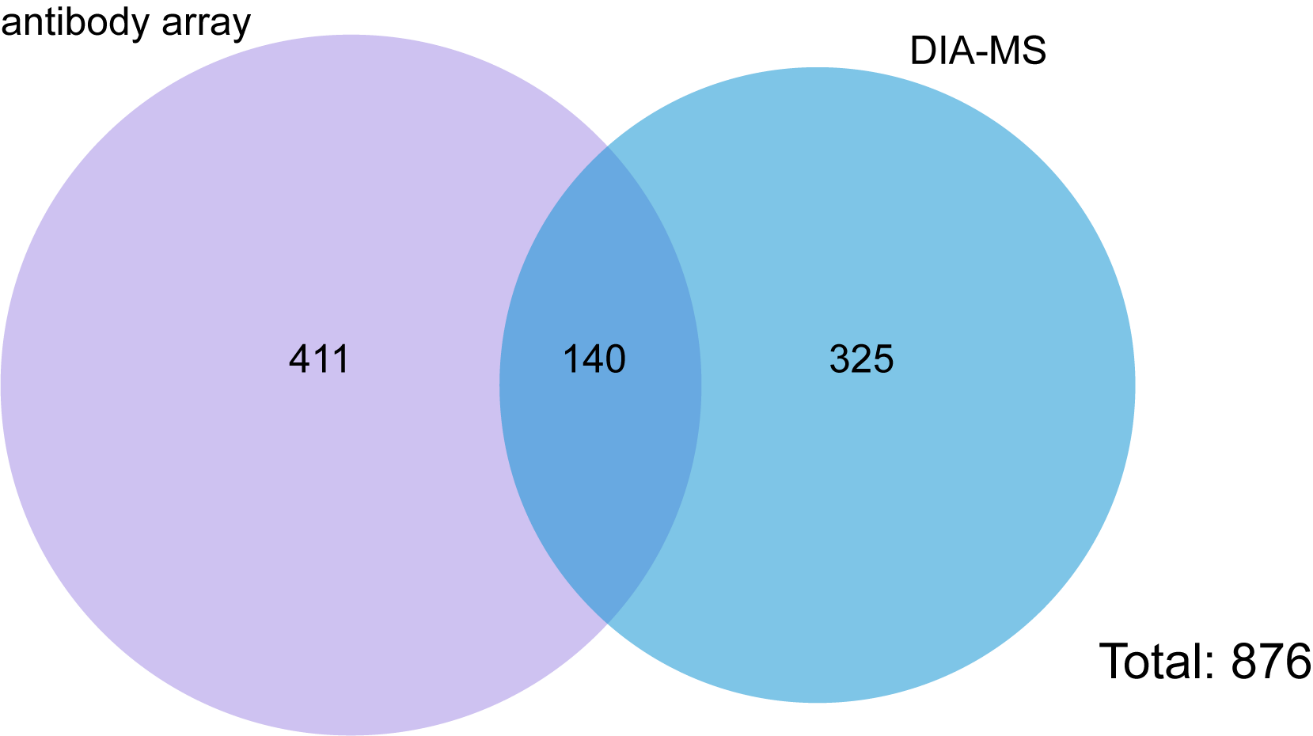
**

**Appendix Figure S3. Venn diagram of proteins identified by antibody array and DIA-MS analyses.**


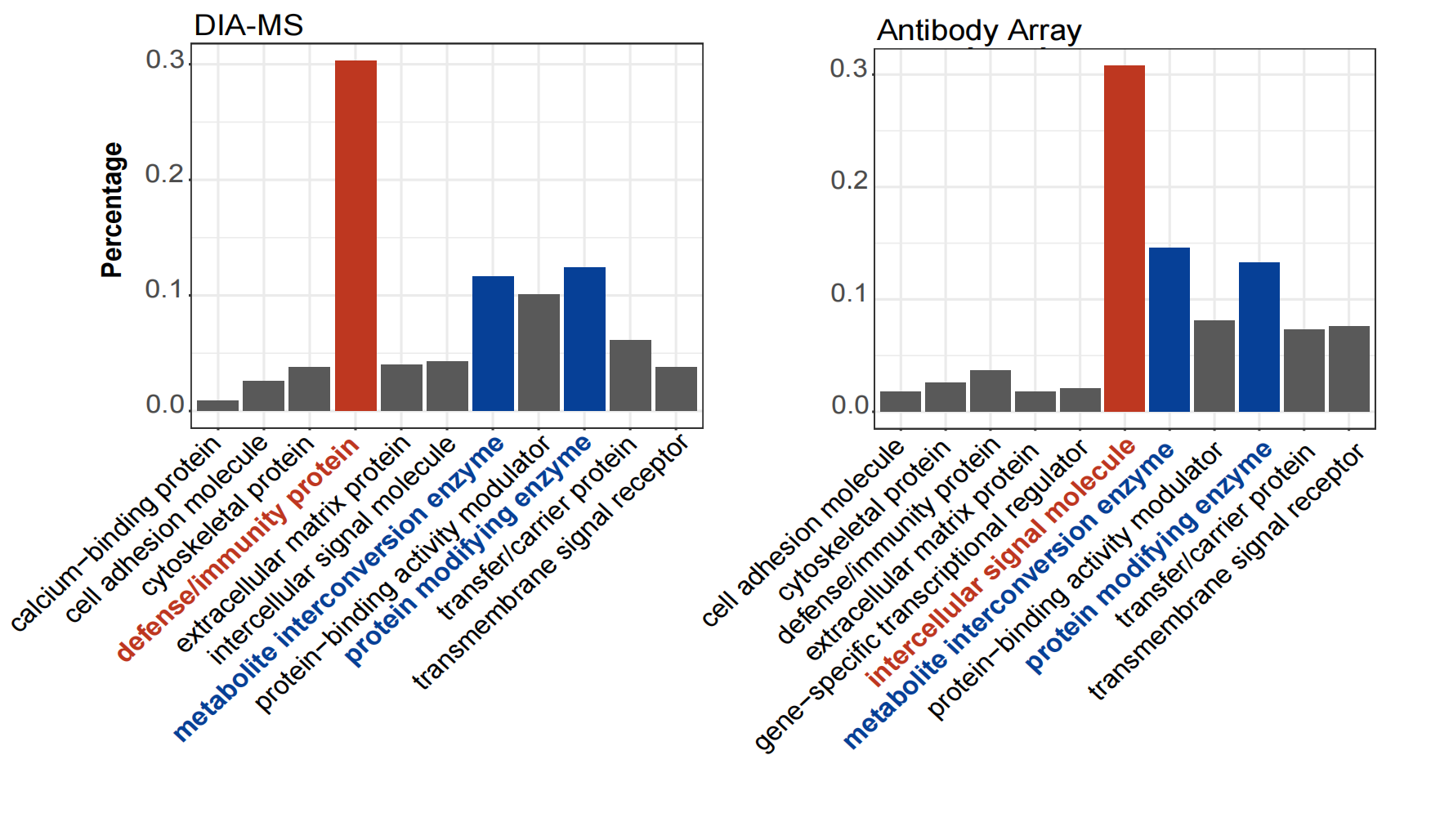


**Appendix Figure S4. Enriched classes of differential plasma proteins in DLBCL patients compared to HCs.** Protein classes were obtained from the PANTHER database. Red indicates proteins elevated in DLBCL while blue color represents proteins elevated in HCs.


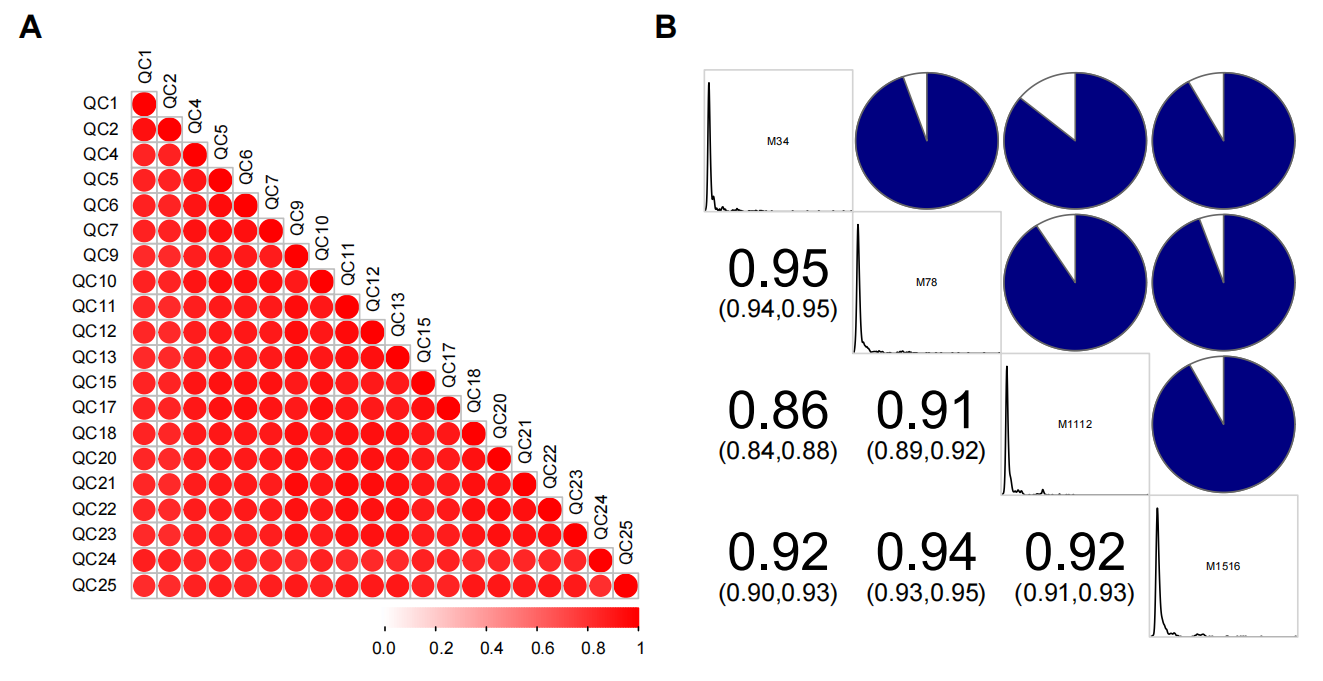


**Appendix Figure S5. Reproducibility of plasma proteome detection using DIA-MS and antibody arrays.**

(A) Reproducibility of DIA-MS detection of plasma proteins.

(B) Reproducibility of microarray detection of plasma proteins.


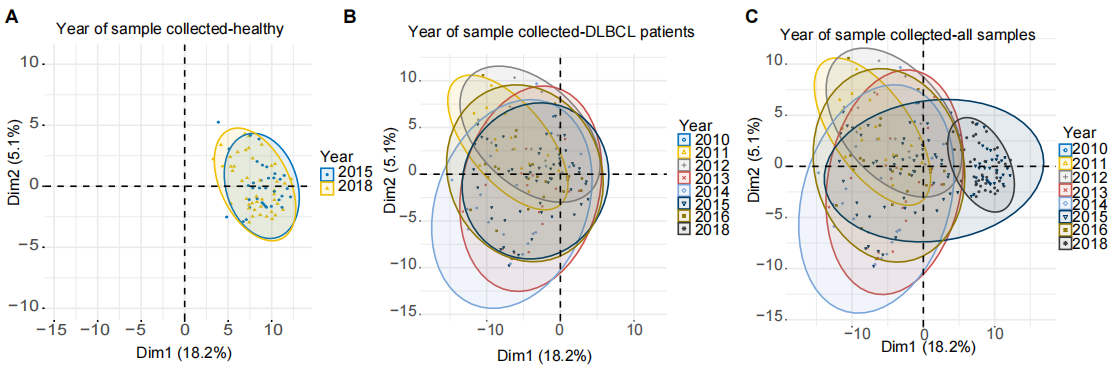


**Appendix Figure S6: PCA plot of samples collected at different times.**

(A-C) are the PCA analysis of plasma samples from HC, DLBCL and both groups, respectively.


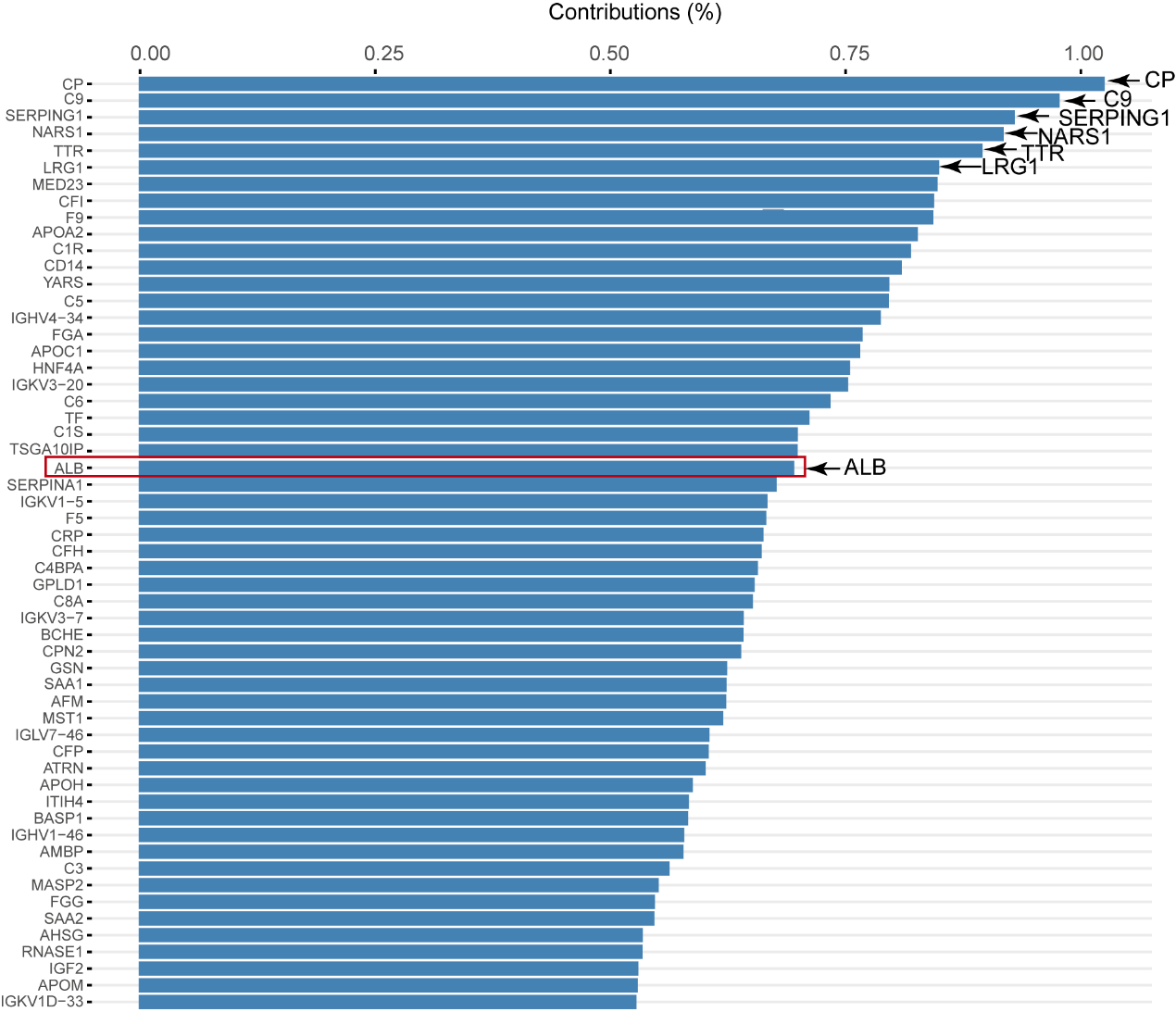


**Appendix Figure S7. Contribution of each variable to the separation of principal component (PC) 1 and PC2 in the PCA.**


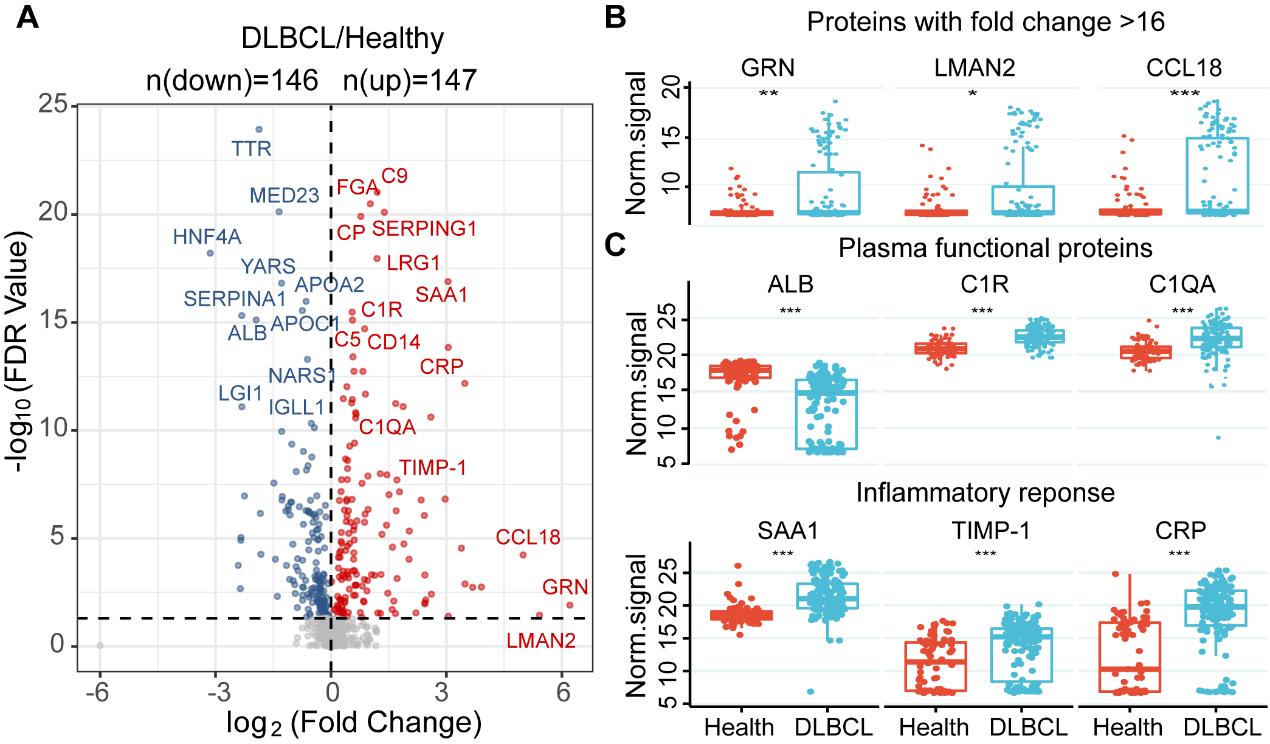


**Appendix Figure S8. Identification of differentially-expressed proteins between DLBCL patients and HCs.**

(A) Volcano plot analysis of differentially expressed proteins. Significantly differential proteins (FDR < 0.05) are highlighted in red (fold change; FC ≥ 1) and blue (FC ＜ 1). Significance is based on FDR-value calculated by the Wilcoxon rank-sum test (two-sided, non-paired).

(B)-(C) Selective presentation of proteins with fold changes above 16, plasma functional and inflammatory response proteins. The dysregulated proteins were identified using the Wilcoxon rank-sum test (two-sided, non-paired) with a FDR < 0.05.


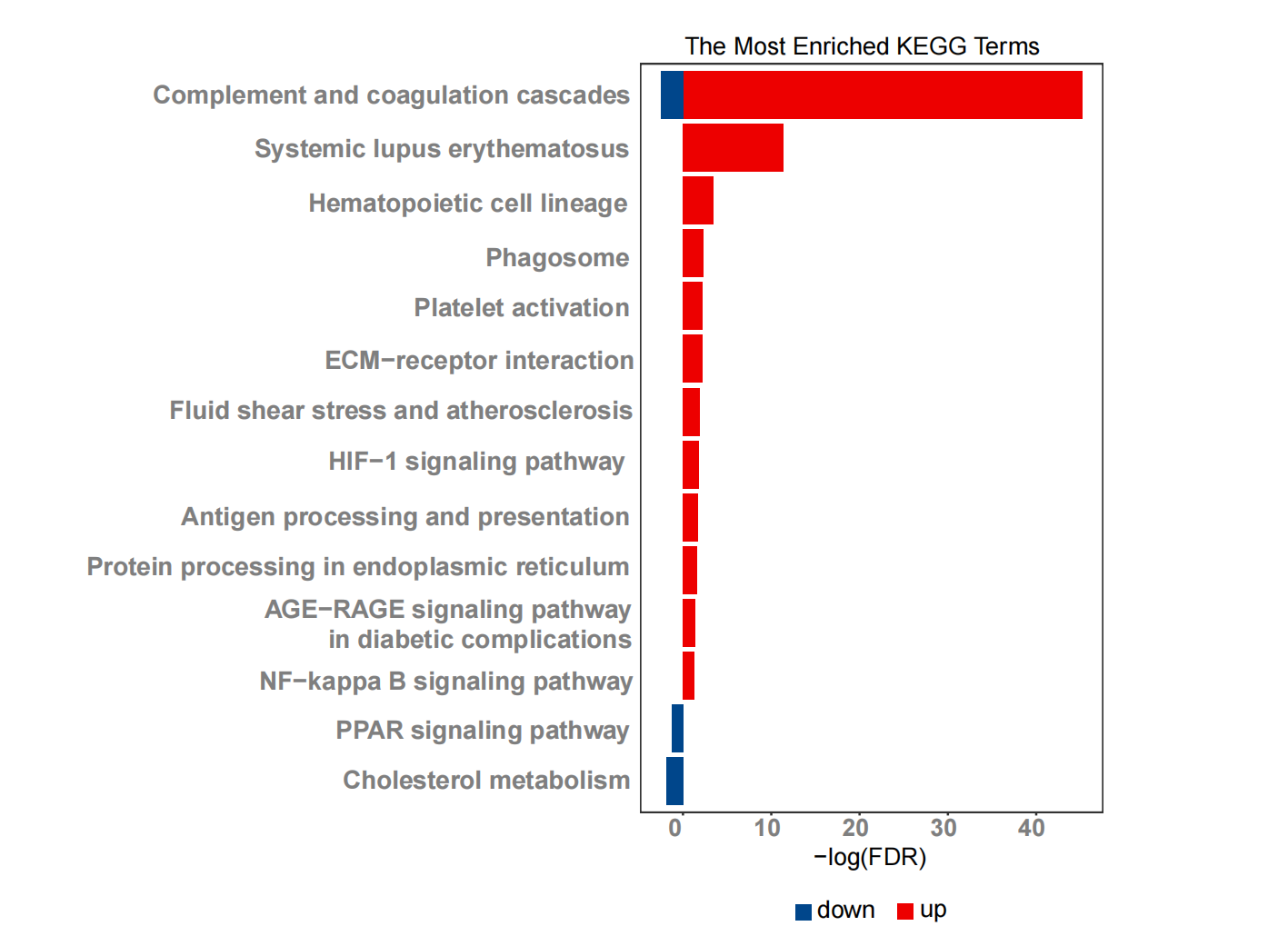


**Appendix Figure S9. Enriched KEGG pathways of dysregulated proteins in the plasma of DLBCL patients compared to HCs.** Enriched pathways had an FDR value < 0.05.


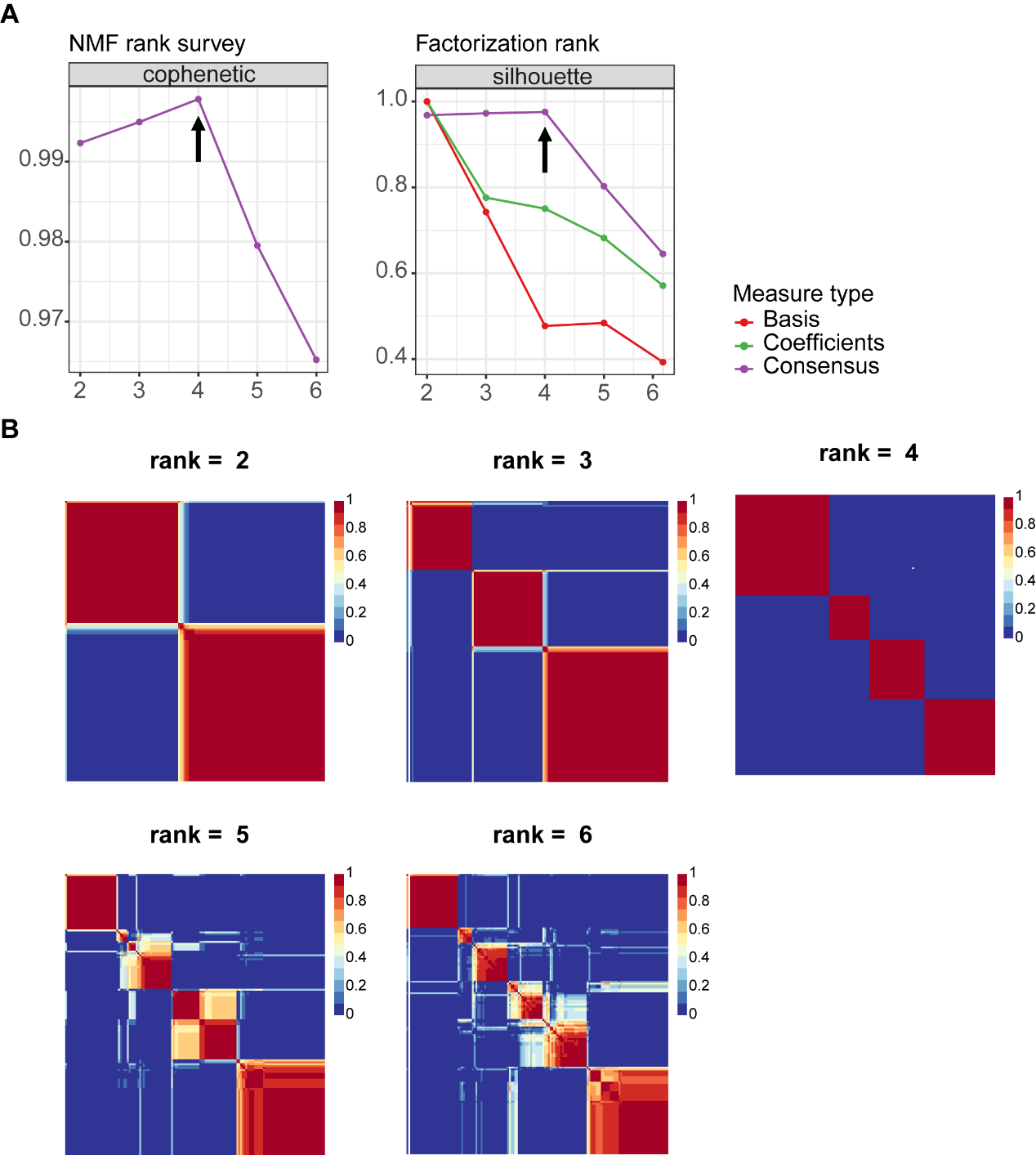


**Appendix Figure S10. NMF quality metrics.**

1. Cophenetic correlation coefficient and average silhouette-width plots suggest rank four (k = 4) gave the best split of the data. The estimation is based on Brunet’s algorithm.
2. Consensus heatmap plots of rank 2-6 suggest rank four (k = 4) gave the most stable split of the data.


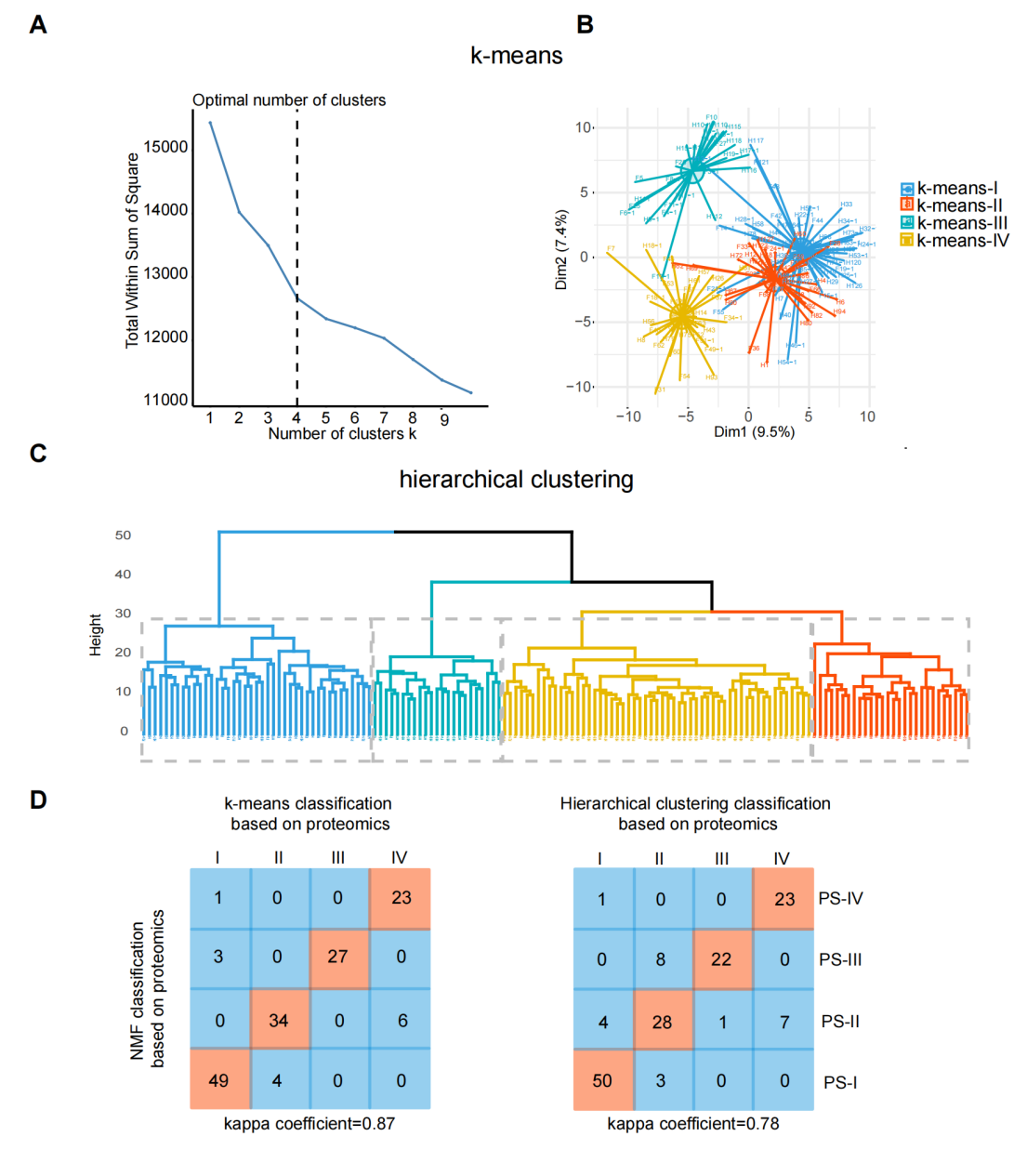


**Appendix Figure S11. DLBCL subtypes identified by k-means or hierarchical clustering methods using proteomic data.**

(A)The sum of the squared distance between each cluster member and its centroid. (B) PCA plot displays the distinction of the four DLBCL subtypes clustered by k-means.

(C) The dendrogram of hierarchical clustering. PS-I cluster (yellow), PS-II cluster (orange), PS-III cluster (green), PS-IV cluster (blue).

(D) The correlation between plasma proteomics subtypes and other classification systems based on other unsupervised clustering methods.


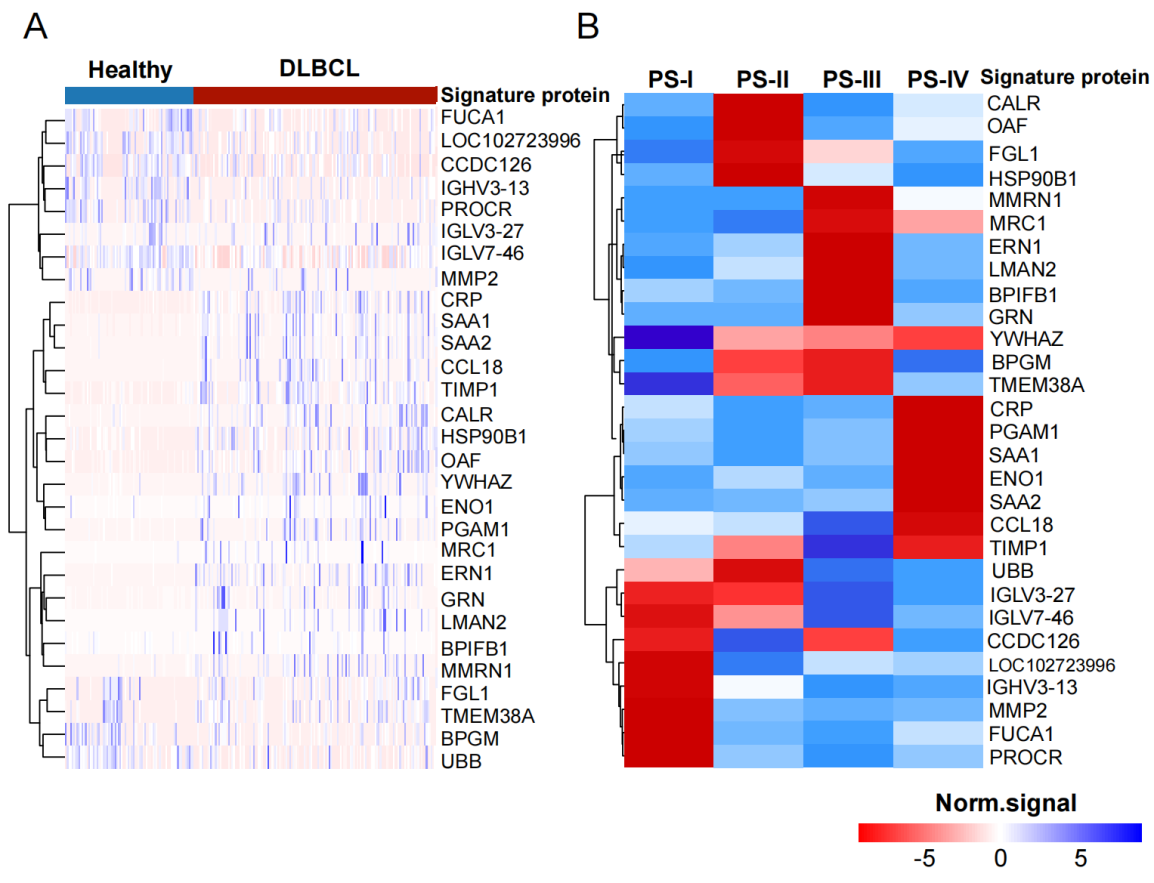


**Appendix Figure S12. Hierachical clustering analysis of signature proteins in the four proteomic subtypes.** (A) and (B) were drawn using the 29 signature proteins with the normalized signal after log2 transformation.

**Appendix Figure S13. Expression of PS-IV proteins in liver tissue.** Enrichment analysis was executed using the STRING database in Cytoscape (version 3.7.2) with an FDR value < 0.05.


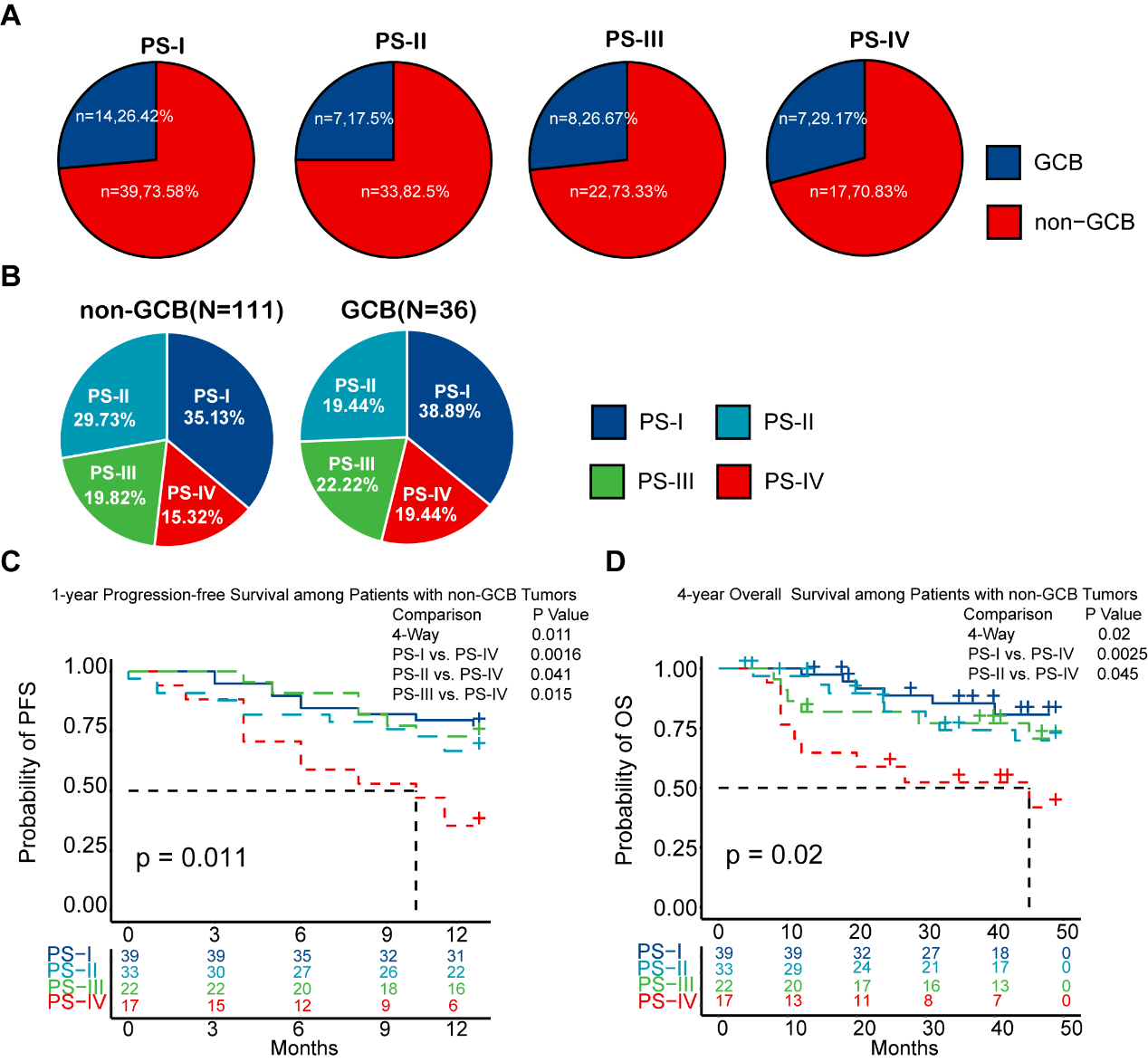


**Appendix Figure S14. Classifying DLBCL patients based on proteomic subtypes or the Hans classification system.**


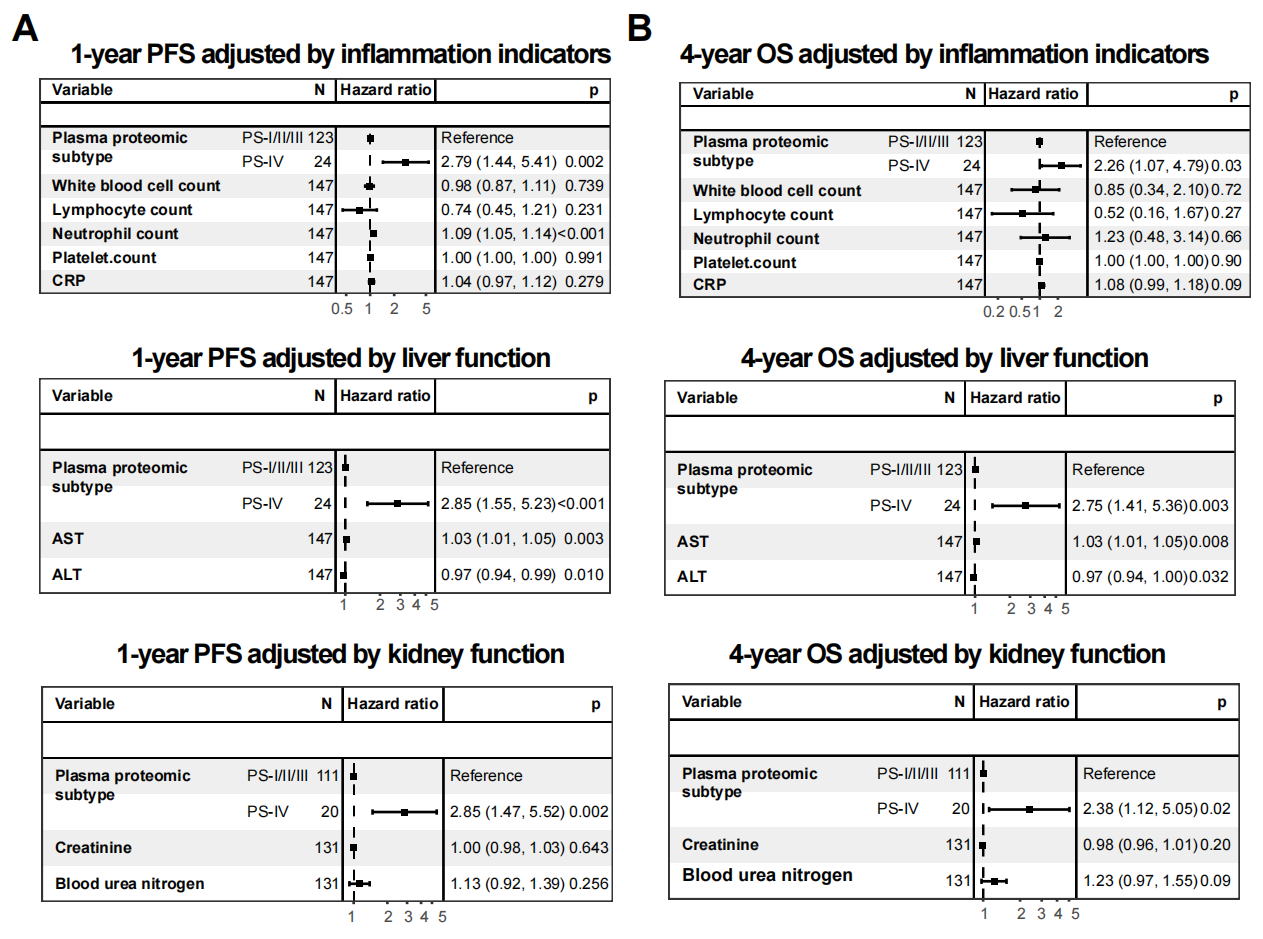


**Appendix Figure S15. The prognostic value of plasma proteomic subtypes adjusted by inflammation, liver and kidney function** **using multivariate Cox analysis.**


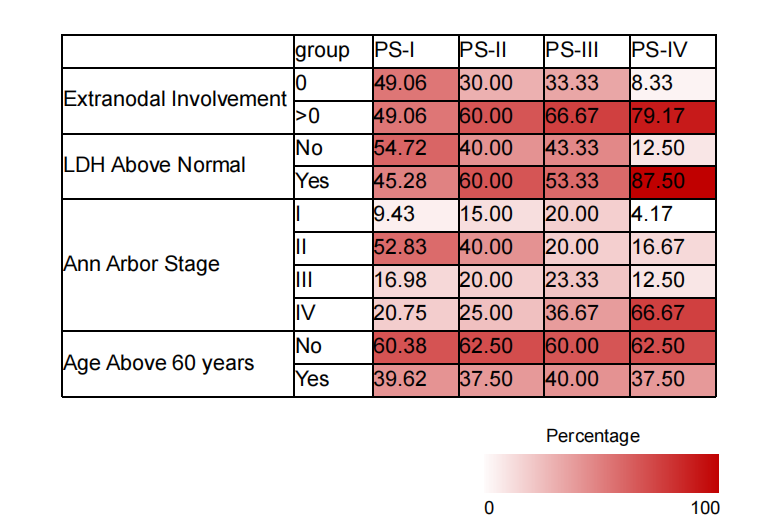


**Appendix Figure S16. Association between plasma proteomic subtypes and the clinical variables.**


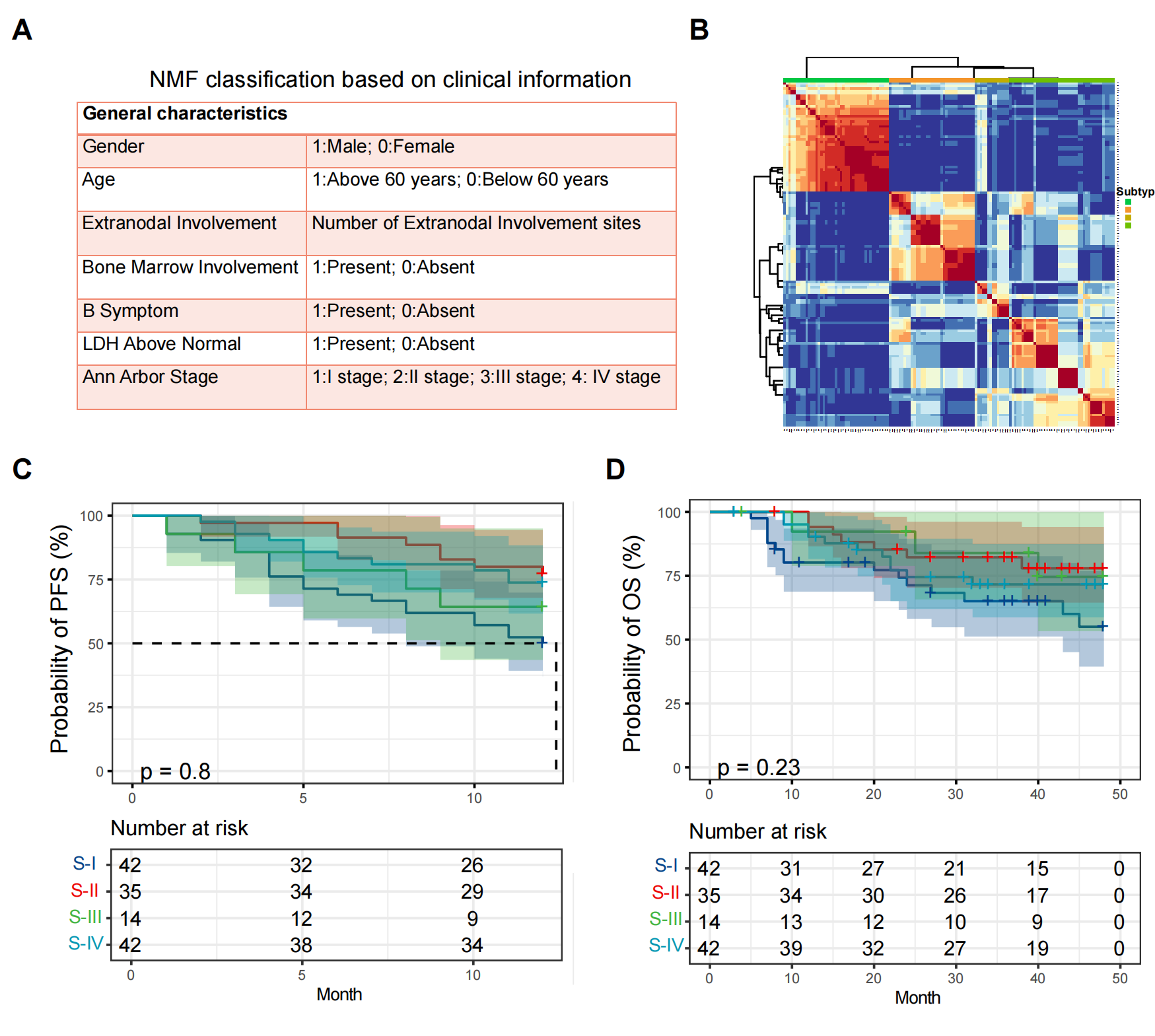


**Appendix Figure S17. DLBCL subtypes identified by clinical data with the NMF method.**

(A) Definitions of clinical variables recorded. LDH: lactate dehydrogenase.

(B) Heatmap of NMF consensus matrix of four subtypes based on clinical variables.

(C) The distinction of 1-year PFS of four clinical subtypes.

(D) The distinction of 4-year OS of four clinical subtypes.


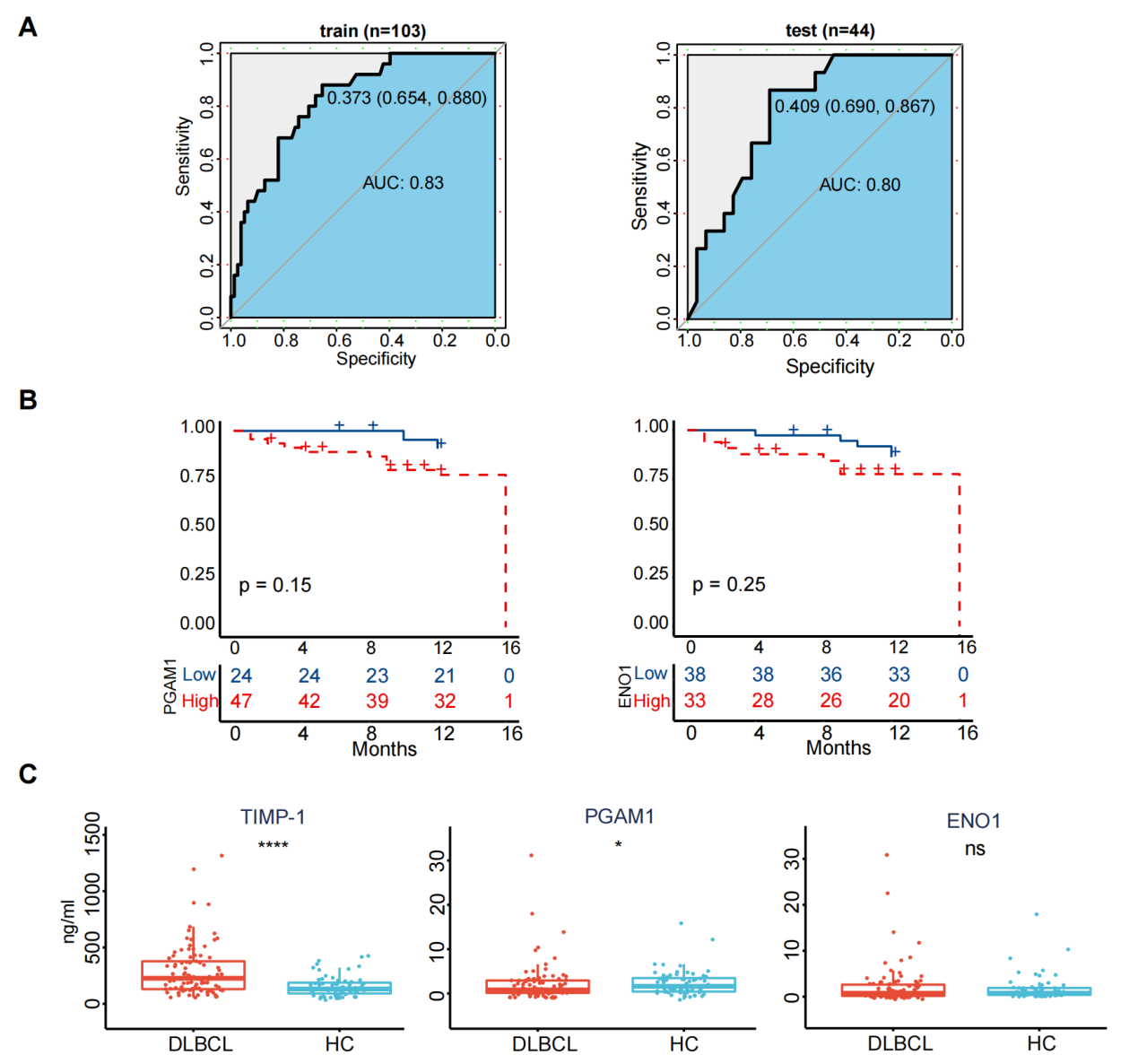


**Appendix Figure S18. The AUC of the subtyping model and the prognostic value of PGAM1 and ENO1.**

(A) The AUC of the subtyping model in the train dataset (n=103) and test dataset (n=44). AUC, area under the curve

(B) The prognostic value of PGAM1 and ENO1. The p value was calculated using the log-rank test.

(C) The comparison between DLBCL patients and healthy controls in validation cohort #1.


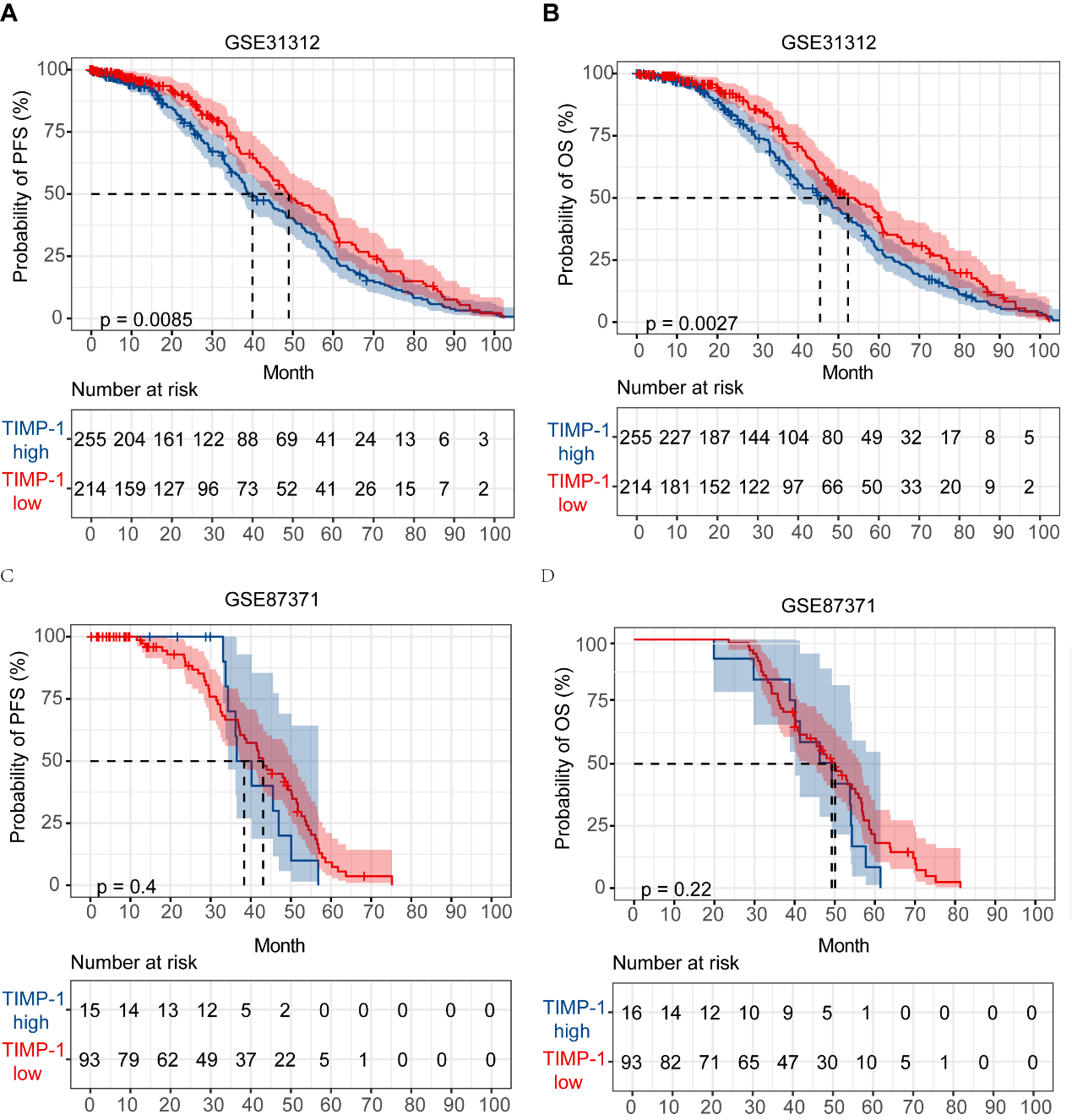


**Appendix Figure S19. The prognostic value of TIMP-1 mRNA using the GSE31312 dataset.** The prognostic value of TIMP-1 mRNA using the GSE31312 dataset. The TIMP-1 mRNA dataset (GSE31312) was obtained from the GEO database (https://www.ncbi.nlm.nih.gov/geo/). "TIMP-1 high" was defined as above the mean value while "TIMP-1 low" was defined as below the mean value. The p value was calculated using the log-rank test.


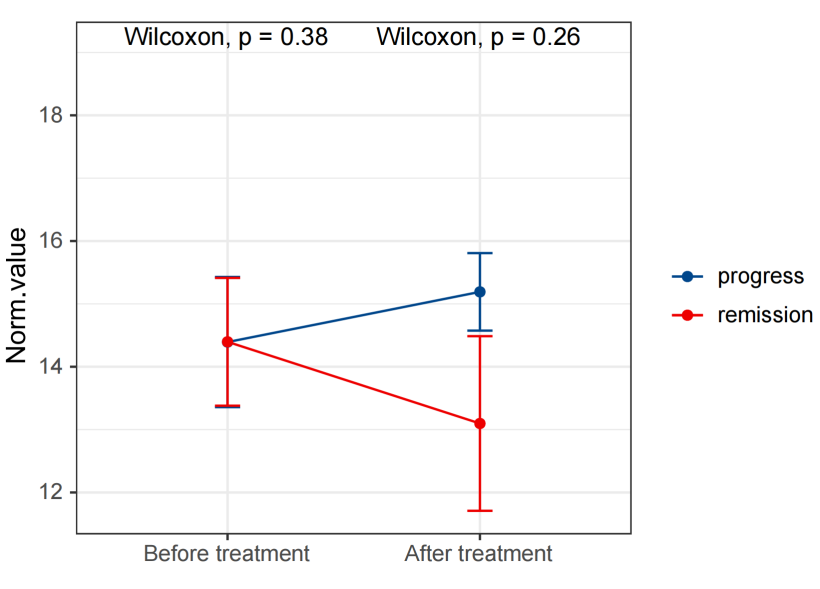


**Appendix Figure S20. Effect of R-CHOP or R-CHOP-like regimens on TIMP-1 level.**


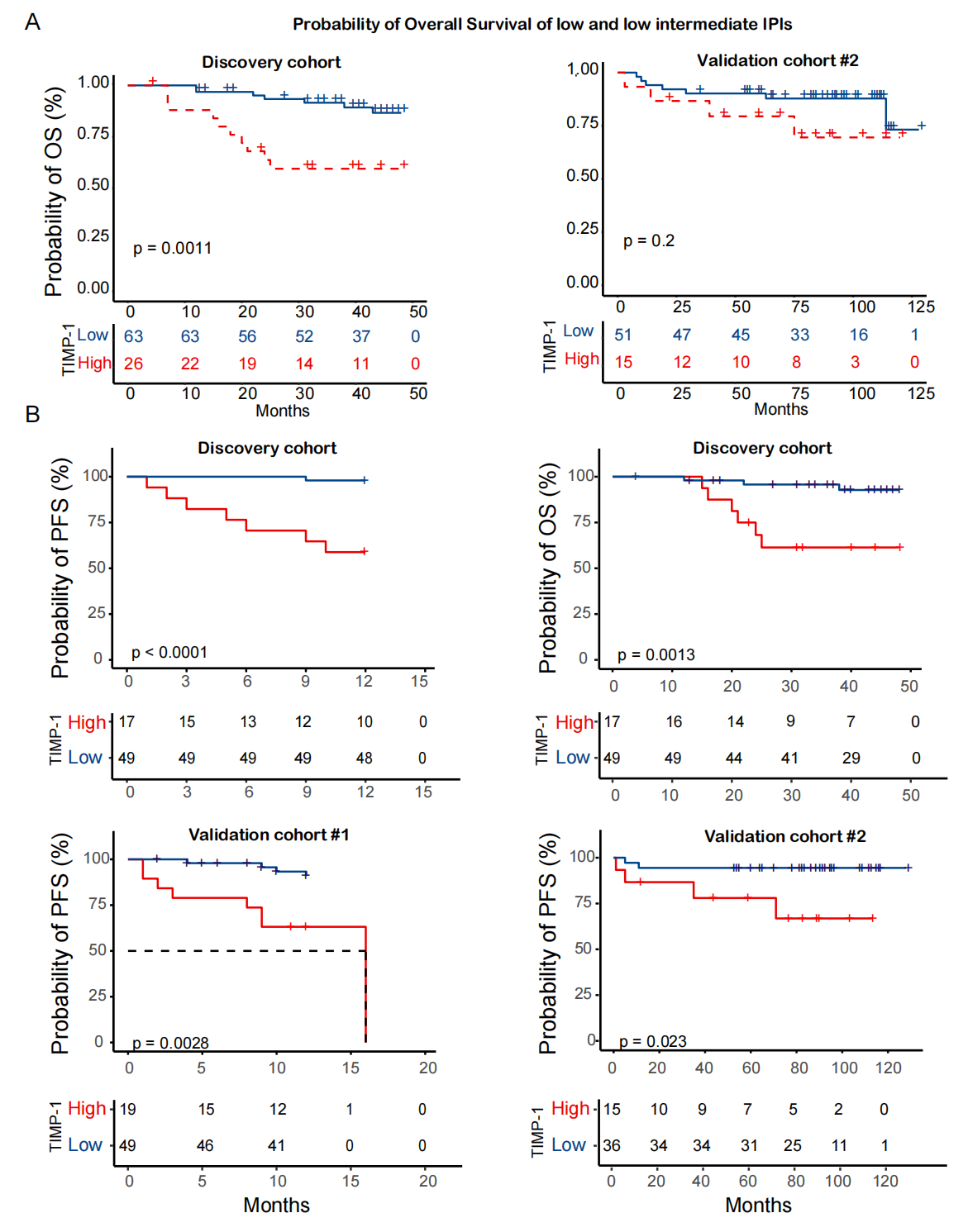


**Appendix Figure S21. Stratification of 4-year OS in DLBCL patients with low-risk IPIs and early-stage based on the level of TIMP-1 in plasma.**

(A) Kaplan-Meier models of 4-year OS within low-risk IPIs (IPI=0,1,2) based on plasma TIMP-1 in the discovery cohort and validation cohort #2.

(B) Kaplan-Meier models of OS and PFS within early-stage DLBCLs (early stage=I,II) based on plasma TIMP-1 in the discovery cohort, validation cohort #1 and #2.

**Appendix Tables**

**Appendix Table S1. Summary of different DLBCL classifications obtained to date.**

|  | IPI score | Transcriptomic subtype system | Hans classification system | Genomic subtype system |
| --- | --- | --- | --- | --- |
| Year | 1993 | 2000，2002 | 2004 | 2018，2020 |
| Clinical utility | Y | N | Y | N |
| Rationale | Age, ECOG score， Ann Arbor stage， Extranodal involvement， LDH | 17，856 genes  Expression | IL-6、CD10、BCL6、MUM1 | 168 gene mutations |
| subtypes | Low, low intermediate, high intermediate, high | GCB， ABC， others | GCB，non-GCB | MCD, BN2, EZB, ST2, A53, N1 |

LDH: lactate dehydrogenase; GCB: germinal-center B-cell-like; ABC: activated B-cell-like

**Appendix Table S6. The list of proteins that differ significantly between DLBCL and HCs detected by both DIA-MS and antibody arrays.**

| Protein | FDR value (AA) | FDR value (DIA-MS) |
| --- | --- | --- |
| APOC4 | 0.022577121 | 1.40066E-05 |
| C1R | 6.58445E-07 | 3.33114E-16 |
| C1S | 0.008447955 | 3.41304E-12 |
| C8A | 0.021083296 | 2.47186E-09 |
| F11 | 1.07335E-06 | 1.53325E-06 |
| F2 | 0.027834262 | 5.28173E-06 |
| FGB | 0.007215896 | 1.55713E-07 |
| FN1 | 0.003860122 | 0.000791479 |
| GPX3 | 0.049421761 | 0.013108331 |
| HP | 0.001418825 | 5.73865E-12 |
| ITIH4 | 0.028098091 | 5.79668E-09 |
| LPA | 0.014322676 | 1.95912E-08 |
| PROCR | 0.049277705 | 0.000177129 |
| SERPINA7 | 0.021277416 | 0.002915415 |
| SERPINC1 | 8.9813E-06 | 1.74172E-07 |
| TNC | 7.4936E-07 | 0.001921561 |

**Appendix Table S7. The list of proteins that differ significantly between early-stage DLBCL and HCs detected by both DIA-MS and antibody arrays**

| Protein | FDR value (AA) | FDR value (DIA-MS) |
| --- | --- | --- |
| C1R | 0.000163132 | 7.57373E-12 |
| C8A | 0.030903808 | 8.18441E-07 |
| F11 | 0.00078258 | 0.000261188 |
| FGB | 0.010739386 | 8.89806E-06 |
| HP | 0.034252395 | 7.82593E-08 |
| SERPINC1 | 0.000823125 | 3.45972E-07 |

**Appendix Table S9. List of plasma proteins with the normalized value in the six DLBCL clusters identified with proteomics.**

| Gene | HCs | Early stage | Advanced stage | Cluster |
| --- | --- | --- | --- | --- |
| TNC | -0.94 | -0.11 | 1.05 | Cluster 1 |
| B2M | -1.02 | 0.04 | 0.98 | Cluster 1 |
| C1QC | -1.03 | 0.06 | 0.97 | Cluster 1 |
| C4BPA | -1.03 | 0.06 | 0.97 | Cluster 1 |
| C7 | -0.93 | -0.12 | 1.06 | Cluster 1 |
| CCL18 | -0.74 | -0.40 | 1.14 | Cluster 1 |
| CD44 | -0.72 | -0.42 | 1.14 | Cluster 1 |
| CP | -1.03 | 0.06 | 0.97 | Cluster 1 |
| CRP | -0.78 | -0.35 | 1.13 | Cluster 1 |
| EFEMP1 | -1.02 | 0.04 | 0.98 | Cluster 1 |
| ERN1 | -1.04 | 0.08 | 0.96 | Cluster 1 |
| FCGR3A | -1.04 | 0.08 | 0.96 | Cluster 1 |
| GRN | -0.90 | -0.18 | 1.08 | Cluster 1 |
| HP | -1.00 | 0.01 | 1.00 | Cluster 1 |
| HPX | -0.90 | -0.17 | 1.07 | Cluster 1 |
| HSP90B1 | -1.01 | 0.02 | 0.99 | Cluster 1 |
| HSPA8 | -0.70 | -0.45 | 1.15 | Cluster 1 |
| ICAM1 | -0.63 | -0.52 | 1.15 | Cluster 1 |
| IGFBP2 | -1.01 | 0.03 | 0.99 | Cluster 1 |
| LBP | -0.91 | -0.16 | 1.07 | Cluster 1 |
| LCP1 | -0.84 | -0.27 | 1.11 | Cluster 1 |
| LRG1 | -0.99 | -0.02 | 1.01 | Cluster 1 |
| MRC1 | -0.95 | -0.10 | 1.04 | Cluster 1 |
| ORM1 | -0.92 | -0.14 | 1.06 | Cluster 1 |
| SAA1 | -0.97 | -0.05 | 1.02 | Cluster 1 |
| SAA2 | -0.90 | -0.17 | 1.07 | Cluster 1 |
| SELL | -1.01 | 0.03 | 0.99 | Cluster 1 |
| SERPINA11 | -1.03 | 0.05 | 0.97 | Cluster 1 |
| SERPINA3 | 0.15 | -1.07 | 0.92 | Cluster 1 |
| TFRC | -0.85 | -0.25 | 1.10 | Cluster 1 |
| TIMP1 | -0.81 | -0.31 | 1.12 | Cluster 1 |
| UBB | 0.15 | -1.07 | 0.92 | Cluster 1 |
| YWHAE | -0.92 | -0.14 | 1.06 | Cluster 1 |
| B4E1Z4 | -0.98 | -0.04 | 1.02 | Cluster 1 |
| A1BG | -1.10 | 0.26 | 0.84 | Cluster 2 |
| ABI3BP | -1.05 | 0.10 | 0.95 | Cluster 2 |
| AGT | -1.13 | 0.37 | 0.76 | Cluster 2 |
| AMBP | -1.15 | 0.47 | 0.68 | Cluster 2 |
| APMAP | -1.12 | 0.32 | 0.80 | Cluster 2 |
| APOE | -1.10 | 0.23 | 0.86 | Cluster 2 |
| BASP1 | -1.06 | 0.14 | 0.92 | Cluster 2 |
| C1QA | -1.08 | 0.18 | 0.90 | Cluster 2 |
| C1S | -1.13 | 0.38 | 0.75 | Cluster 2 |
| C2 | -1.10 | 0.24 | 0.86 | Cluster 2 |
| C3 | -1.15 | 0.45 | 0.70 | Cluster 2 |
| C5 | -1.14 | 0.39 | 0.75 | Cluster 2 |
| C8A | -1.14 | 0.41 | 0.73 | Cluster 2 |
| C8B | -1.09 | 0.23 | 0.87 | Cluster 2 |
| C8G | -1.14 | 0.39 | 0.75 | Cluster 2 |
| C9 | -1.10 | 0.25 | 0.85 | Cluster 2 |
| CALR | -1.11 | 0.27 | 0.84 | Cluster 2 |
| CD14 | -1.11 | 0.29 | 0.82 | Cluster 2 |
| CD163 | -1.14 | 0.39 | 0.74 | Cluster 2 |
| CFH | -1.14 | 0.39 | 0.75 | Cluster 2 |
| CFHR2 | -1.08 | 0.19 | 0.89 | Cluster 2 |
| CFHR5 | -1.10 | 0.26 | 0.85 | Cluster 2 |
| CFI | -1.12 | 0.31 | 0.81 | Cluster 2 |
| CPN2 | -1.14 | 0.42 | 0.72 | Cluster 2 |
| F11 | -1.06 | 0.14 | 0.92 | Cluster 2 |
| F5 | -1.15 | 0.44 | 0.70 | Cluster 2 |
| F9 | -1.13 | 0.35 | 0.78 | Cluster 2 |
| FGA | -1.13 | 0.34 | 0.79 | Cluster 2 |
| FGB | -1.15 | 0.45 | 0.69 | Cluster 2 |
| FGG | -1.06 | 0.13 | 0.93 | Cluster 2 |
| HABP2 | -1.13 | 0.37 | 0.77 | Cluster 2 |
| HSPA5 | -1.11 | 0.29 | 0.82 | Cluster 2 |
| IGHM | -1.13 | 0.36 | 0.77 | Cluster 2 |
| ITIH4 | -1.13 | 0.36 | 0.77 | Cluster 2 |
| KRT6B | -1.13 | 0.36 | 0.77 | Cluster 2 |
| MAN1A1 | -1.11 | 0.28 | 0.83 | Cluster 2 |
| MASP2 | -1.10 | 0.23 | 0.86 | Cluster 2 |
| MST1 | -1.13 | 0.35 | 0.78 | Cluster 2 |
| PIGR | -1.09 | 0.23 | 0.87 | Cluster 2 |
| PLG | -1.12 | 0.33 | 0.79 | Cluster 2 |
| PRG4 | -1.15 | 0.45 | 0.70 | Cluster 2 |
| RNASE1 | -1.15 | 0.48 | 0.67 | Cluster 2 |
| SERPING1 | -1.15 | 0.47 | 0.68 | Cluster 2 |
| VCL | -1.12 | 0.32 | 0.80 | Cluster 2 |
| VTN | -1.15 | 0.44 | 0.70 | Cluster 2 |
| YWHAZ | -1.12 | 0.31 | 0.81 | Cluster 2 |
| ZGRF1 | -1.13 | 0.36 | 0.77 | Cluster 2 |
| APOB | 0.93 | -1.06 | 0.13 | Cluster 3 |
| MMP1 | 1.11 | -0.84 | -0.27 | Cluster 3 |
| NT5C2 | 1.06 | -0.92 | -0.14 | Cluster 3 |
| PPIA | 1.14 | -0.73 | -0.41 | Cluster 3 |
| S3 | 1.15 | -0.61 | -0.55 | Cluster 3 |
| S4 | 1.08 | -0.90 | -0.18 | Cluster 3 |
| S5 | 1.15 | -0.64 | -0.51 | Cluster 3 |
| SERPINF2 | 0.43 | -1.14 | 0.71 | Cluster 3 |
| APOC4-APOC2 | 1.15 | -0.51 | -0.64 | Cluster 3 |
| AZGP1 | 1.15 | -0.52 | -0.64 | Cluster 3 |
| BLVRB | 1.15 | -0.60 | -0.55 | Cluster 3 |
| BPGM | 1.15 | -0.67 | -0.48 | Cluster 3 |
| CA1 | 1.15 | -0.70 | -0.44 | Cluster 3 |
| CA2 | 0.81 | -1.12 | 0.30 | Cluster 3 |
| CAMP | 1.15 | -0.62 | -0.53 | Cluster 3 |
| CAT | 1.15 | -0.61 | -0.54 | Cluster 3 |
| FHL3 | 1.15 | -0.68 | -0.46 | Cluster 3 |
| FN1 | 1.15 | -0.55 | -0.61 | Cluster 3 |
| HBA1 | 1.14 | -0.75 | -0.39 | Cluster 3 |
| HBA2 | 1.07 | -0.91 | -0.17 | Cluster 3 |
| HBB | 1.14 | -0.72 | -0.42 | Cluster 3 |
| HBD | 1.15 | -0.61 | -0.55 | Cluster 3 |
| HNF4A | 1.15 | -0.67 | -0.48 | Cluster 3 |
| IGHV1-45 | 1.15 | -0.61 | -0.54 | Cluster 3 |
| IGHV1-8 | 1.15 | -0.69 | -0.46 | Cluster 3 |
| IGKV2-29 | 1.15 | -0.59 | -0.56 | Cluster 3 |
| IGKV3-7 | 1.15 | -0.48 | -0.67 | Cluster 3 |
| IGKV4-1 | 1.11 | -0.82 | -0.29 | Cluster 3 |
| IGLC2 | 0.71 | -1.14 | 0.43 | Cluster 3 |
| IGLV1-51 | 1.15 | -0.64 | -0.52 | Cluster 3 |
| IGLV3-9 | 1.14 | -0.74 | -0.40 | Cluster 3 |
| LAMP2 | 1.01 | -0.99 | -0.03 | Cluster 3 |
| LDHB | 0.38 | -1.13 | 0.76 | Cluster 3 |
| PNP | 1.15 | -0.61 | -0.54 | Cluster 3 |
| PODXL | 1.15 | -0.62 | -0.53 | Cluster 3 |
| PRDX1 | 1.13 | -0.77 | -0.36 | Cluster 3 |
| PRDX2 | 1.14 | -0.73 | -0.41 | Cluster 3 |
| PROCR | 1.15 | -0.61 | -0.54 | Cluster 3 |
| RAN | 0.93 | -1.06 | 0.13 | Cluster 3 |
| SERPINA1 | 1.14 | -0.74 | -0.39 | Cluster 3 |
| SLC4A1 | 1.14 | -0.72 | -0.42 | Cluster 3 |
| TMEM38A | 1.15 | -0.49 | -0.66 | Cluster 3 |
| GAS6 | -1.15 | 0.68 | 0.47 | Cluster 4 |
| IL1A | -1.15 | 0.64 | 0.51 | Cluster 4 |
| APOH | -1.12 | 0.81 | 0.30 | Cluster 4 |
| ATRN | -1.13 | 0.75 | 0.38 | Cluster 4 |
| C1R | -1.15 | 0.56 | 0.59 | Cluster 4 |
| C1RL | -1.15 | 0.51 | 0.65 | Cluster 4 |
| C4A | -1.15 | 0.63 | 0.52 | Cluster 4 |
| C4B | -1.15 | 0.58 | 0.58 | Cluster 4 |
| C6 | -1.15 | 0.61 | 0.54 | Cluster 4 |
| CETP | -1.15 | 0.66 | 0.49 | Cluster 4 |
| CFP | -1.15 | 0.66 | 0.49 | Cluster 4 |
| CPB2 | -1.13 | 0.76 | 0.38 | Cluster 4 |
| F10 | -1.15 | 0.63 | 0.53 | Cluster 4 |
| F2 | -1.12 | 0.79 | 0.33 | Cluster 4 |
| FCN2 | -1.15 | 0.65 | 0.50 | Cluster 4 |
| KNG1 | -1.15 | 0.64 | 0.51 | Cluster 4 |
| LPA | -1.15 | 0.50 | 0.65 | Cluster 4 |
| OAF | -1.15 | 0.66 | 0.49 | Cluster 4 |
| PTPRJ | -1.11 | 0.82 | 0.29 | Cluster 4 |
| RARRES2 | -1.15 | 0.69 | 0.46 | Cluster 4 |
| SERPINC1 | -1.14 | 0.75 | 0.39 | Cluster 4 |
| TF | -1.11 | 0.83 | 0.28 | Cluster 4 |
| TGFBI | -1.12 | 0.80 | 0.33 | Cluster 4 |
| CXCL5 | 1.14 | -0.42 | -0.72 | Cluster 5 |
| EDN1 | 1.09 | -0.21 | -0.88 | Cluster 5 |
| A2M | 1.11 | -0.27 | -0.84 | Cluster 5 |
| AHSG | 1.00 | 0.00 | -1.00 | Cluster 5 |
| ALB | 1.15 | -0.45 | -0.69 | Cluster 5 |
| APCS | 0.62 | 0.54 | -1.15 | Cluster 5 |
| APOA2 | 1.05 | -0.11 | -0.94 | Cluster 5 |
| APOC1 | 1.11 | -0.29 | -0.82 | Cluster 5 |
| APOC4 | 1.13 | -0.38 | -0.76 | Cluster 5 |
| APOM | 1.12 | -0.33 | -0.79 | Cluster 5 |
| BCHE | 0.94 | 0.12 | -1.05 | Cluster 5 |
| CLU | 1.14 | -0.41 | -0.73 | Cluster 5 |
| CNDP1 | 1.09 | -0.21 | -0.88 | Cluster 5 |
| CRTAC1 | 1.04 | -0.08 | -0.96 | Cluster 5 |
| FGL1 | 0.36 | 0.77 | -1.13 | Cluster 5 |
| GSN | 1.03 | -0.06 | -0.97 | Cluster 5 |
| IGF2 | 1.04 | -0.08 | -0.96 | Cluster 5 |
| IGHG2 | 1.15 | -0.45 | -0.69 | Cluster 5 |
| IGHG4 | 1.12 | -0.31 | -0.81 | Cluster 5 |
| IGHV1-46 | 1.13 | -0.36 | -0.77 | Cluster 5 |
| IGHV3-13 | 1.12 | -0.31 | -0.81 | Cluster 5 |
| IGHV3-7 | 1.12 | -0.31 | -0.81 | Cluster 5 |
| IGHV3-72 | 1.12 | -0.30 | -0.81 | Cluster 5 |
| IGHV4-34 | 1.10 | -0.25 | -0.85 | Cluster 5 |
| IGKV1-5 | 1.11 | -0.28 | -0.83 | Cluster 5 |
| IGKV1D-33 | 1.10 | -0.24 | -0.86 | Cluster 5 |
| IGKV2D-29 | 1.11 | -0.28 | -0.83 | Cluster 5 |
| IGKV3-20 | 1.12 | -0.33 | -0.79 | Cluster 5 |
| IGLC7 | 1.13 | -0.37 | -0.76 | Cluster 5 |
| IGLL1 | 0.56 | 0.59 | -1.15 | Cluster 5 |
| IGLL5 | 1.12 | -0.31 | -0.81 | Cluster 5 |
| IGLV3-10 | 1.09 | -0.21 | -0.88 | Cluster 5 |
| IGLV3-25 | 1.07 | -0.16 | -0.91 | Cluster 5 |
| IGLV5-45 | 1.10 | -0.24 | -0.86 | Cluster 5 |
| IGLV7-46 | 1.05 | -0.11 | -0.94 | Cluster 5 |
| IGLV8-61 | 1.14 | -0.39 | -0.75 | Cluster 5 |
| KRTDAP | 1.14 | -0.42 | -0.72 | Cluster 5 |
| LGI1 | 1.11 | -0.27 | -0.84 | Cluster 5 |
| LOC102723996 | 1.14 | -0.41 | -0.73 | Cluster 5 |
| MCAM | 1.15 | -0.45 | -0.69 | Cluster 5 |
| MED23 | 1.14 | -0.43 | -0.71 | Cluster 5 |
| MMP2 | 1.02 | -0.05 | -0.97 | Cluster 5 |
| NARS1 | 1.10 | -0.24 | -0.86 | Cluster 5 |
| PI16 | 1.01 | -0.02 | -0.99 | Cluster 5 |
| SELENOP | 1.11 | -0.27 | -0.84 | Cluster 5 |
| SERPINA4 | 1.03 | -0.07 | -0.96 | Cluster 5 |
| SERPINA7 | 1.11 | -0.29 | -0.82 | Cluster 5 |
| TSGA10IP | 1.08 | -0.19 | -0.89 | Cluster 5 |
| TTR | 1.15 | -0.45 | -0.70 | Cluster 5 |
| YARS | 1.15 | -0.45 | -0.69 | Cluster 5 |
| ITGB3 | -1.04 | 0.95 | 0.09 | Cluster 6 |
| PTH | -0.95 | 1.05 | -0.10 | Cluster 6 |
| APOA4 | -0.87 | 1.09 | -0.23 | Cluster 6 |
| CFD | -1.08 | 0.90 | 0.18 | Cluster 6 |
| GC | -1.07 | 0.91 | 0.15 | Cluster 6 |
| HLA-C | -0.95 | 1.04 | -0.09 | Cluster 6 |
| IGFALS | -0.55 | 1.15 | -0.61 | Cluster 6 |
| IGHV3-73 | -0.95 | 1.04 | -0.10 | Cluster 6 |
| IGKV2-30 | -0.95 | 1.05 | -0.10 | Cluster 6 |
| INHBC | -1.06 | 0.92 | 0.14 | Cluster 6 |
| KRT5 | -0.74 | 1.14 | -0.40 | Cluster 6 |
| KRT9 | -1.07 | 0.91 | 0.16 | Cluster 6 |
| LTF | -1.02 | 0.97 | 0.05 | Cluster 6 |
| PCOLCE | -1.01 | 0.99 | 0.01 | Cluster 6 |
| PRSS2 | -0.98 | 1.02 | -0.04 | Cluster 6 |
| VASN | -0.98 | 1.02 | -0.04 | Cluster 6 |
